# Peroxisomal interactome mapping enables network-based modelling of function and disease

**DOI:** 10.26508/lsa.202503562

**Published:** 2026-07-01

**Authors:** Søren W Gersting, Julia V Cramer, Philipp Guder, Amelie S Lotz-Havla, Barbara Wolf, Heidi Noll-Puchta, Ralf Erdmann, Ania C Muntau, Mathias Woidy

**Affiliations:** 1 University Children’s Research, UCR@Kinder-UKE, University Medical Center Hamburg-Eppendorf, Hamburg, Germany; 2 German Center for Child and Adolescent Health (DZKJ), Partner Site Hamburg, University Medical Center Hamburg-Eppendorf (UKE), Hamburg, Germany; 3 University Children’s Hospital, University Medical Center Hamburg-Eppendorf , Hamburg, Germany; 4 Dr. von Hauner Children’s Hospital, LMU University Hospital, Ludwig-Maximilians-Universität München , Munich, Germany; 5 Children’s Hospital, Klinikum Dritter Orden Munich, Munich, Germany; 6 Ruhr-Universität Bochum, Medical Faculty, Institute of Biochemistry and Pathobiochemistry, Systems Biochemistry, Bochum, Germany

## Abstract

Peroxisomal dysfunction contributes to a broad spectrum of multisystem disorders, yet mechanistic understanding and therapeutic options remain limited, posing significant challenges for clinical management. Network-based computational strategies support hypothesis generation, biomarker discovery, and drug repurposing, but their usage is constrained by incomplete human interactome coverage—especially by scarcity of high-confidence protein–protein interaction (PPI) data for peroxisomal proteins. We present the first comprehensive map of the peroxisomal interactome, generated using an automated, informatics-guided bioluminescence resonance energy transfer strategy. We profiled PPIs for 92 peroxisomal proteins and six isoforms, validating 68% of known interactions and identifying 333 novel ones. Integration with curated PPIs yielded an expanded peroxisomal interactome, enriched for drug targets and disease-associated proteins. A disease-linked subnetwork enabled prioritization of drug repurposing candidates. Tissue-specific expanded peroxisomal interactome variants, derived from transcriptomic data, revealed distinct functional submodules across nine tissues. Gene ontology analysis of 1,272 non-peroxisomal interactors suggested pathways contributing to tissue-specific vulnerability. Our approach provides a systems-level framework for mechanistic insight in peroxisomal disease, the identification of treatment targets, and application to other organelle systems.

## Abstract (precis)

Filling a critical gap in the human interactome, we define a comprehensive peroxisomal interaction network that enables network-based hypothesis generation for tissue-specific function, disease mechanisms, and therapeutic exploration.

## Introduction

Peroxisomes are small organelles that play numerous roles in human metabolism, such as fatty acid breakdown, bile acid and sterol synthesis, and amino-acid metabolism (Wanders et al, 2023). In recent years, it has become increasingly clear that peroxisomes are central organelles with functions beyond metabolism, including roles in cancer development, neurodegenerative disease such as Alzheimer’s disease, and innate immune response (Zalckvar & Schuldiner, 2022). They interact closely with other organelles, including mitochondria, lipid droplets, and the endoplasmic reticulum (Wanders et al, 2016). Peroxisomes consist of structural proteins involved in biogenesis and maintenance (peroxins), as well as functional proteins required for enzymatic processes and transport (Wanders et al, 2023).

Peroxisomal biogenesis disorders (PBDs) belong to the group of inborn errors of metabolism and are rare diseases caused by genetic defects in peroxins. Pathogenic variants in these genes result in a broad spectrum of diseases (Klouwer et al, 2015). The severity ranges from mild forms with late-onset symptoms, such as enamel problems, to severe multisystemic forms with early lethality, such as Zellweger syndrome. Pathogenic variants in non-PBD genes can lead to similar phenotypes with varying clinical presentations (Waterham et al, 2015). Among these, *ABCD1* is the most frequently affected non-PBD gene, causing X-linked adrenoleukodystrophy (X-ALD) (Kemp et al, 2016), a disease that ranges from mild to severe, with the latter presenting progressive multisystemic symptoms, including cognitive decline, blindness, deafness, and adrenal failure. Peroxisomal functions in health and disease are not fully understood (Wanders et al, 2023), and as a result, therapeutic options are mostly limited to supportive care. As an exception, in X-ALD, hematopoietic stem cell therapy is a therapeutic option in some patients (Engelen et al, 2012). Further research is needed to improve our understanding of the physiological functions of peroxisomes and the diseases associated with them.

Cellular functions are governed by a multitude of macromolecular interactions, collectively referred to as the human interactome. The more we understand the human interactome—particularly the interplay of different cellular components—the better we will understand the molecular basis of cellular function and dysfunction (Luck et al, 2020). In recent years, efforts have been made to systematically map protein interactions, leading to the generation of large-scale reference maps (Luck et al, 2020; Huttlin et al, 2021). In addition to these large-scale maps, smaller, more focused maps—such as those of specific cellular regions like cilia (Boldt et al, 2016), the centrosome (Galletta et al, 2016), mitochondria (Antonicka et al, 2020), or the interactomes of individual proteins like ATP7A (Comstra et al, 2017) — have provided additional insights into cellular protein interactions. The increasing resolution of the human interactome offers new network-based methods for exploring the molecular complexity of human diseases (Menche et al, 2015; Caldera et al, 2017; Silverman et al, 2020). Network medicine approaches leverage the fact that biological data, such as the human interactome, can be represented as a graph, in which nodes represent proteins and edges denote interactions. One key paradigm in network medicine is the idea that diseases can be viewed as local perturbations within the network (Barabási et al, 2011; Silverman et al, 2020). Another paradigm suggests that proteins associated with the same disease are likely to interact with one another (Barabási et al, 2011; Menche et al, 2015; Silverman et al, 2020). Network medicine has been successfully applied to a wide range of diseases, including COPD (Sharma et al, 2018), metabolic syndrome (Misselbeck et al, 2019), axonopathies (Bis-Brewer et al, 2019), and Fabry disease (Braun et al, 2023). We have demonstrated its applicability to inborn errors of metabolism (Woidy et al, 2018). However, because network-based approaches depend on the topology of the underlying networks, the results of such analyses are influenced by the completeness of the data, and further mapping efforts are required (Menche et al, 2015; Luck et al, 2020; Silverman et al, 2020).

Applying network-based approaches to study peroxisomal diseases is challenging due to the lack of peroxisomal protein interaction data. In this study, we used our developed iBRET approach to experimentally map a set of peroxisomal interactions (Lotz-Havla et al, 2021a). By integrating these with literature-based interactions, the resulting network expands our understanding of the peroxisomal interactome. We demonstrate that this network can be used to apply network-based approaches and generate new hypotheses about the molecular perturbations underlying peroxisomal diseases, leading to tailored hypothesis-driven experimental workups.

## Results

### Mapping the peroxisomal interactome by means of iBRET

**Figure 1.**
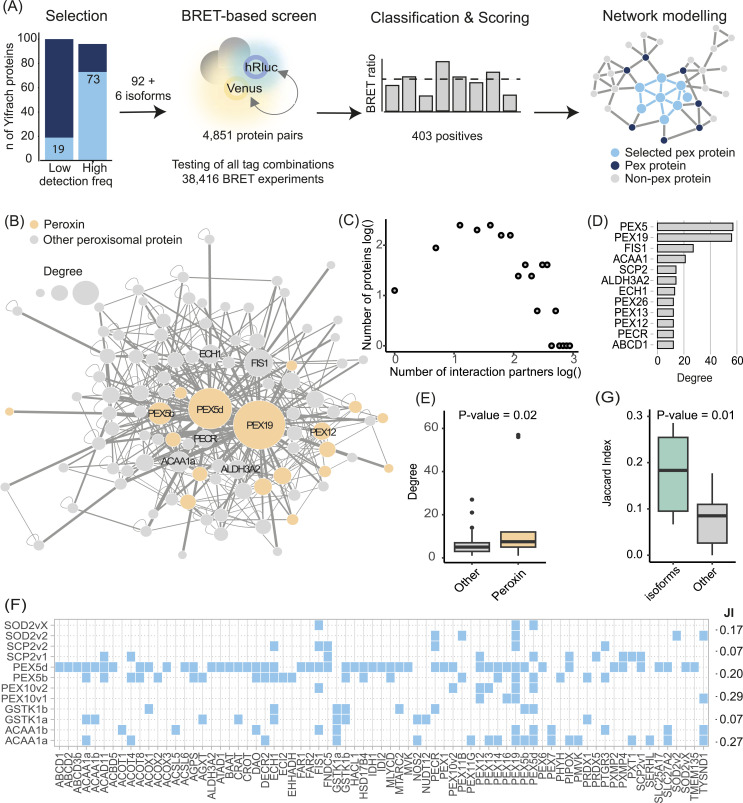
Mapping the peroxisomal interactome. **(A)** Schematic overview of our approach. **(B)** Network representation of the peroxisomal interactome. Nodes represent peroxisomal (pex) proteins and edges PPI detected by our BRET-based screening method. Peroxins are colored in orange. The size of a node depicts the respective degree of the protein. **(C)** Degree distribution of our peroxisomal interactome. **(D)** Degree of the top hub proteins of the peroxisomal interactome. **(E)** Degree of peroxins versus other peroxisomal proteins. Peroxins have a higher average degree than all other peroxisomal proteins. **(F)** Heatmap of protein interactions of the included pairs of isoforms. A blue-colored rectangle represents a protein interaction found by our BRET-based screen. On the right y-axis the Jaccard Index (JI) for each pair of isoforms is depicted (see Materials and Methods section for further details of calculation). **(G)** Comparison of the pairwise JI of isoforms against other peroxisomal proteins. Isoforms more often share interaction partners than pairs of other peroxisomal proteins.

First, to experimentally map peroxisomal PPIs, we searched our in-house protein library for peroxisomal proteins, based on recently published data by Yifrach et al (2018). This search yielded 73 proteins with high detection frequency and 19 proteins with low detection frequency for peroxisomal localization, giving a total of 92 proteins. Overall, we included 76% of all proteins with high detection frequency from the Yifrach dataset. For 6 of the 92 proteins, we investigated an additional isoform, resulting in a total of 98 proteins (Fig 1A, Supplemental Data 1).

Supplemental Data 1. Overview of the included peroxisomal genes.

Second, to systematically screen each of the possible 4,851 protein pairs for interactions, we applied our recently described informatics-aided protein interaction (PPI) mapping platform, which is based on bioluminescence resonance energy transfer (iBRET) (Lotz-Havla et al, 2021a) (Fig 1A). In total, we conducted 38,416 iBRET experiments. Automated classification identified 403 interacting peroxisomal pairs (Fig 1B, Supplemental Data 2).

Supplemental Data 2. Interacting peroxisomal protein pairs.

**Figure S1.**
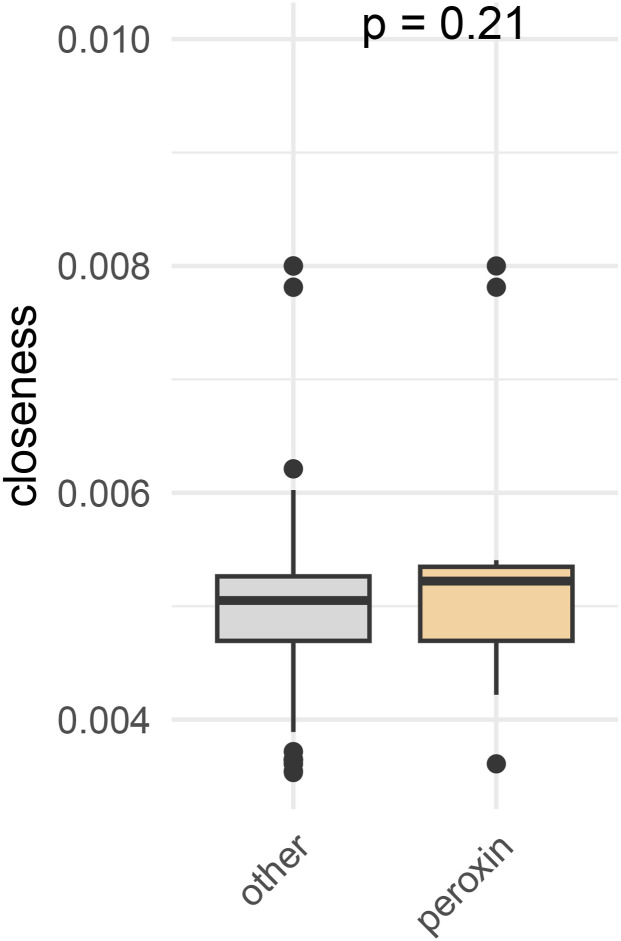
eness centrality of peroxins versus other peroxisomal proteins. Both groups do not significantly differ. Clos

**Figure S2.**
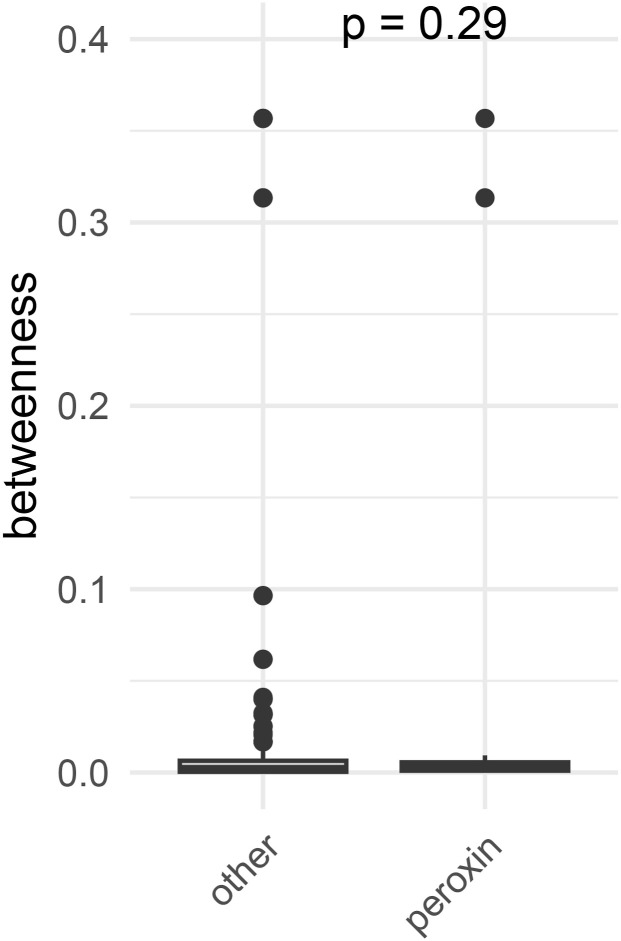
Betweenness centrality of peroxins versus other peroxisomal proteins. Both groups do not significantly differ.

The degree distribution of our experimentally defined peroxisomal interactome (PI) is consistent with heavy-tailed behavior and cannot be distinguished from a log–normal or power-law distribution based on likelihood-ratio testing (Fig 1C, power-law versus log–normal R = −0.680, *P*-value = 0.50, see the Materials and Methods section). Heavy-tailed distributions, specifically power-law distributions, are a typical feature of biological networks and suggest that a few highly connected hub proteins are central to the integrity of the entire network (Caldera et al, 2017). Among the hubs in the PI, the peroxins PEX5 and PEX19 exhibit the highest number of mapped interactions (Fig 1D). Additionally, peroxins generally display a higher average degree of connectivity compared to other peroxisomal proteins (*P*-value = 0.02, Wilcoxon-rank test, Fig 1E), underscoring their importance for peroxisomal biogenesis and protein import (Kumar et al, 2024). However, other centrality measures, such as closeness centrality (cc) and betweenness centrality (bc), did not show significant differences between peroxins and other peroxisomal proteins (see Figs S1 and S2). Interestingly, the third most interactions were found for fission protein 1 (FIS1), a protein that is dually localized to mitochondria and peroxisomes and important for the fission of these organelles (Fig 1D) (Carmichael et al, 2022).

Next, we investigated the binding patterns of the included isoforms. Previous studies have demonstrated that isoforms often share fewer than half of their interaction partners and tend to behave more like distinct proteins rather than variants of one another (Yang et al, 2016). To assess how similar the binding patterns of our included peroxisomal isoforms are, we calculated the Jaccard Index (JI) for each of the six pairs of isoforms. The JI measures the similarity of the interaction partners between two isoforms, where a JI of 1 indicates complete overlap of interaction partners, and a JI of 0 indicates no shared partners. We found the highest JI-values for isoforms of PEX10 (0.29), ACCA1 (0.27), and PEX5 (0.20) (Fig 1F). Contrary to previous findings from Yang et al (2016), we found that our isoforms share interaction partners more frequently than other non-related protein pairs (average JI 0.18 versus 0.08, *P*-value = 0.01, Wilcoxon-rank test) (Fig 1G). Our result could be an artifact of the import machinery (interactions with PEX5, PEX7, and PEX19). However, after removing PPIs with PEX19, PEX5, and PEX7, the JI is 0.10 for isoform pairs and 0.04 for all other non-isoform pairs and still differs significantly (*P*-value = 0.02, Wilcoxon-rank test).

### Expanding the peroxisomal interactome

**Figure 2.**
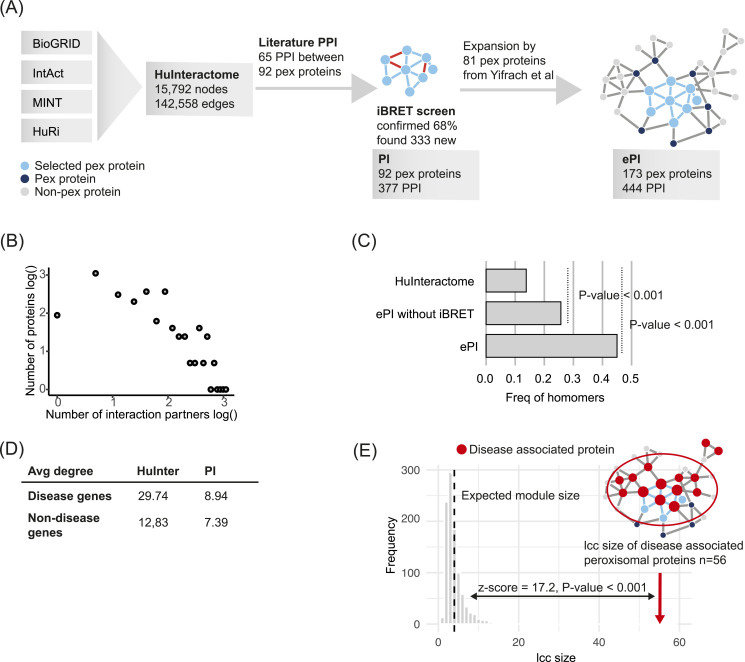
Expanding the peroxisomal interactome. **(A)** Schematic overview of the construction of the peroxisomal interactome (PI) and the expanded peroxisomal interactome (ePI) including previously reported peroxisomal proteins. **(B)** Degree distribution of the extended peroxisomal interactome. **(C)** Comparison of the frequency of homomers in the HuInteractome, the ePI and the ePI without our newly found protein interactions (ePI without iBRET). The ePI is significantly enriched in homomers. **(D)** Comparison of the average degree in peroxisomal disease genes and non-disease genes. Disease genes were derived from the HPO Database. **(E)** Disease associated peroxisomal proteins build a large disease module, measured by the lcc. A z-score was determined by comparing the lcc of the disease module to 1,000 random permutations of lcc calculations of protein sets of the same size.

To compare the PI with known peroxisomal PPIs, we compiled a comprehensive database-based human interactome (HuInteractome), comprising 142,558 experimentally validated PPIs between 15,792 proteins (Fig 2A, Supplemental Data 3). This dataset includes 65 known PPIs among the 92 peroxisomal proteins from our screening. Of these, we confirmed 68% (n = 44) and identified 333 novel peroxisomal interactions using iBRET.

Supplemental Data 3. HuInteractome.

**Figure S3.**
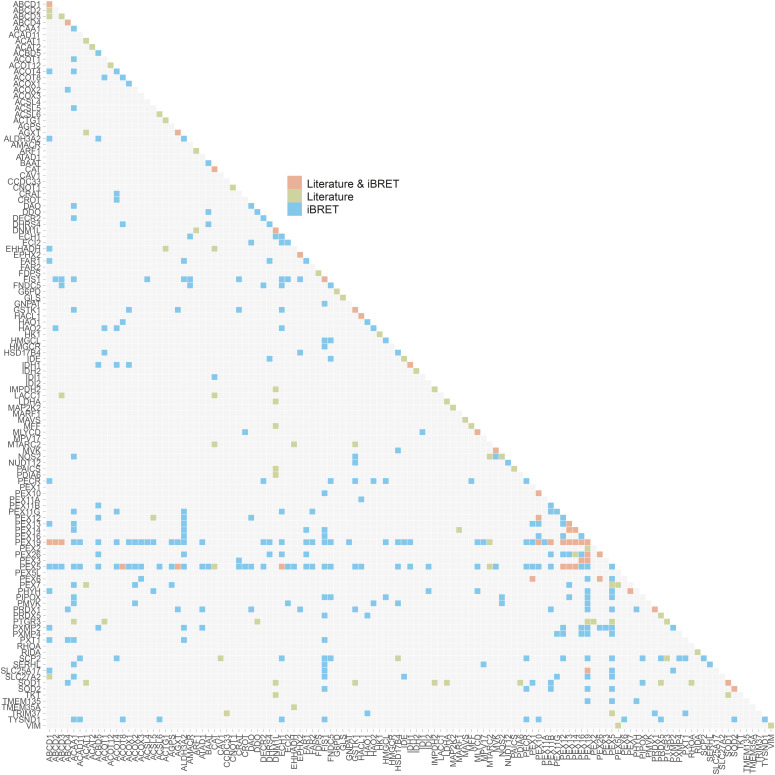
Heatmap representation of the peroxisomal interactions, the color depicts the source the interaction was derived from.

**Figure S4.**
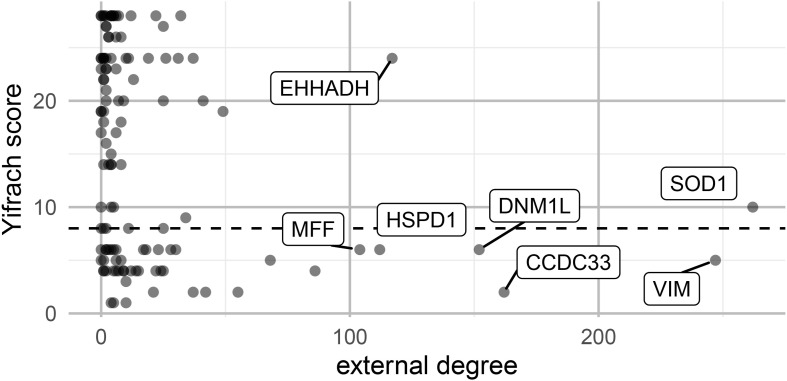
Number of PPI (degree) versus Yifrach score for ePI members. Only the degree to non-ePI members is shown on the x-axis (external degree) enabling the identification of proteins that are more connected to non-ePI members than to ePI members.

For further network-based analyses, we extended the PI to better reflect physiological conditions. To this end, we added peroxisomal proteins from the Yifrach dataset that were not part of our initial experimental setup (see the Materials and Methods section). We constructed the extended peroxisomal interactome (ePI) by combining all previously described peroxisomal interactions from the HuInteractome with the in-house measured interactions from the PI. The ePI comprises 173 peroxisome-associated proteins connected by 444 interactions (Fig S3) and again follows a heavy-tailed distribution (Fig 2B, power-law versus log–normal R = −0.250, *P*-value = 0.80). Of the 173 proteins, 109 form a large, connected component (lcc). The average degree is lower in the ePI compared to the PI and the HuInteractome (5.13 versus 8.20 versus 18.05, median:3 versus 6 versus 7, *P*-value < 0.001, Kruskal–Wallis), indicating that the ePI is more sparsely connected. This is also reflected in the higher number of connected components (ncc) in the ePI (64 versus 1 versus 45) (Table 1). This underscores the lack of peroxisomal PPI in the literature. However, when considering not only the PPIs between ePI members themselves but also their interactions within the entire HuInteractome, the average degree of ePI members increases to 21.53 (median 10). This rise is primarily due to some ePI members having more interactions with non-ePI proteins. These proteins predominantly exhibit low Yifrach scores, suggesting a lower likelihood of peroxisomal localization (see Fig S4). From a network perspective, these proteins might function as connectors between the ePI and the broader cellular interactome.

**Table 1.** Main network characteristics of the human interactome (HuInteractome), the peroxisomal interactome (PI), and the extended peroxisomal interactome (ePI).

​	HuInteractome	PI	ePI
Nodes	15,792	92	173
Edges	142,558	377	444
ncc	45	1	64
N of lcc	15,736	92	109
Avg degree	18.05	8.20	5.13
Freq of disease genes	0.27	0.52	0.51
Freq of drug targets	0.13	0.30	0.32

Given the importance of peroxisomes in metabolic processes and the observation that homo-oligomerization is critical for many metabolic pathways and enzyme activities in general (Hashimoto & Panchenko, 2010), we analyzed whether the ePI is enriched for homooligomers. Indeed, we found a significantly higher proportion of proteins forming homooligomers in the ePI than in the HuInteractome (45%, 78/173, *P*-value < 0.001, Fisher) (Fig 2C). Even after excluding self-interactions identified in our systematic mapping via iBRET, the ePI remained significantly enriched for homomers (27%, 47/173, *P*-value < 0.001, Fisher).

When comparing the average degree of disease-associated proteins with non-disease-associated proteins in the entire HuInteractome, we find the average degree of disease-associated proteins to be significantly higher (29.7 versus 12.8, median: 14 versus 7.5, *P*-value < 0.001, Mann–Whitney *U*). This is a well-known phenomenon referred to as the study bias of disease-associated proteins (Luck et al, 2020). By contrast, due to the unbiased nature of our pairwise iBRET screen, the average degree of disease-associated and non-disease-associated proteins in the ePI does not differ significantly (8.94 versus 7.39, median: 6 versus 6, *P*-value = 0.41 Mann–Whitney *U*) (Fig 2D). We also find that the ePI is enriched in disease-associated proteins when compared to the HuInteractome (51% versus 27%, *P*-value < 0.001, Fisher) and contains more proteins known to be a drug target than the HuInteractome (32% versus 13%, *P*-value < 0.001, Fisher) (Table 1). Based on the paradigm that proteins associated with the same disease are likely to interact with one another and build a disease module (Menche et al, 2015), we calculated whether disease-associated proteins from the ePI form a disease module. We support this hypothesis by finding that 56 out of 89 disease-associated proteins build a module that is larger than the random expectation (ePI-disease module, n = 56, z-score = 17.207, empirical *P*-value < 0.001) (Fig 2E). To evaluate whether the disease module connectivity depends on literature-derived interactions, we reconstructed the module using only BRET-derived edges. In this network, 43 of the 48 disease-associated proteins remained connected through 143 BRET interactions. In contrast, a module constructed solely from literature-derived PPIs connected only 33 of the 88 disease-associated proteins with 60 edges, indicating that the disease module connectivity is largely supported by the experimentally derived BRET interaction network. In summary, the network-based characteristics of the ePI mirroring known pathophysiology make it a robust foundation for further computational investigations into peroxisomal function.

### Network-based drug repurposing

Network-based drug repurposing represents a rapid and cost-effective strategy to identify potential drug candidates for the treatment of specific diseases. This approach relies on the proximity principle, which posits that a drug is more likely to be effective if its targets are located near a disease module within the human interactome (Guney et al, 2016; Cheng et al, 2019). To identify potential therapeutic candidates for peroxisomal disorders, we applied this principle and calculated the network-based distances between 2,186 drugs (based on their known target proteins) and the ePI disease module. The drugs were ranked based on the observed z-score obtained from 1,000 degree-preserved random drawings (see the Materials and Methods section and Supplemental Data 4). Among the top candidates is Taurine, a non-proteinogenic aminosulfonic acid, which has been implicated in protecting against oxidative stress (Baliou et al, 2021) and has shown neuroprotective effects in models of Alzheimer’s disease (Jangra et al, 2024). Notably, other drugs within this group, such as Huperzine A and Netarsudil, have also been proposed as treatment options for Alzheimer’s disease (Friedli & Inestrosa, 2021; Zheng et al, 2025). Given the established role of peroxisomes in reactive oxygen species metabolism (Schrader & Fahimi, 2006) and their involvement in the pathogenesis of neurodegenerative diseases like Alzheimer’s (Xu et al, 2024), our findings may reflect a close mechanistic link between peroxisomal disorders and Alzheimer’s disease. This overlap highlights a promising opportunity for cross-disease drug repurposing.

Supplemental Data 4. Drug candidates ranked by z-score.

### Integration of tissue-specific expression data

**Figure 3.**
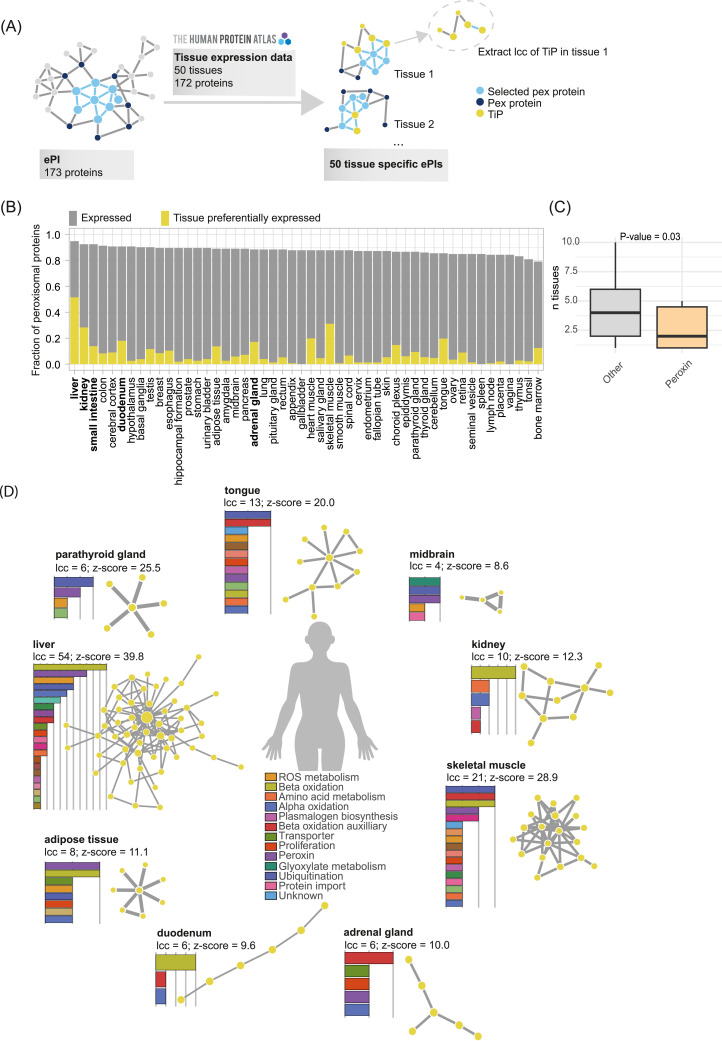
Integration of expression data. **(A)** Schematic overview of the integration of tissue-specific expression data from the HPA database. **(B)** Fraction of expressed and tissue preferentially expressed peroxisomal proteins for each tissue investigated. **(C)** Comparison of tissue-preferential expression frequencies between peroxins and other peroxisomal proteins. Peroxins were significantly less frequently preferentially expressed. *P*-value = 0.03, Mann–Whitney *U* Test. **(D)** Depiction of the tissue-specific lccs of the peroxisomal TiP proteins and their functional association. Lcc sizes of all tissues were larger than compared to random.

While often being multisystemic, peroxisomal dysfunction impact different tissues to a different extent (Zalckvar & Schuldiner, 2022). Especially, adrenal gland, brain, spinal cord, muscle, and retina are affected in peroxisomal diseases. To address this observation, we generated tissue-specific ePIs by integrating expression data from 50 tissues. Expression data were available for 172 of the 173 proteins from the ePI (Fig 3A, see the Materials and Methods section). Of all tissues, the liver expresses the largest number of peroxisomal proteins (n = 163). Moreover, 49 of the 50 tissues express at least 80% of peroxisomal proteins (Fig 3B). Altogether, the majority (=69% [118/172]) of peroxisomal proteins are expressed in all 50 tissues. Only 10 proteins are expressed in fewer than 10 tissues (Supplemental Data 5). Of the latter, none is essential for peroxisomal biogenesis but plays role in different metabolic pathways. The rather uniform expression of peroxisomal proteins led us to the conclusion that it is difficult to use protein expression alone to make statements about tissue-specific functions of peroxisomes. Indeed, Luck and colleagues suggested that tissue-specific functions can rather be explained by tissue-specific expression of proteins alone but by interactions between tissue-preferentially expressed proteins (TiP) and more uniformly expressed proteins (Luck et al, 2020). Hence, detecting such interactions might also help to explain tissue-specific phenotypes and could give insights into tissue-specific function and dysfunction of peroxisomes. We calculated which of the peroxisomal proteins are also TiP and found that 159 of the 172 proteins are at least once a TiP. The 18 proteins that are most often TiP (TiP in ≥7 tissues) are mostly enzymes, and none is a peroxin (Supplemental Data 5). On the other hand, from the 13 proteins that are never a TiP, two are important for peroxisomal biogenesis (PEX12, PEX5L, Supplemental Data 5).

Supplemental Data 5. Tissue expression of peroxisomal proteins.

Indeed, the number of times a peroxisomal protein is tissue-specific was significantly lower for peroxins compared to other peroxisomal proteins (mean 2.73 versus 3.99 versus 4, *P*-value = 0.03 Wilcox, Fig 3C). Peroxisomal TiP was found in 47 of the 50 tissues. Liver, skeletal muscle, kidney, heart muscle, and tongue have the highest number of peroxisomal TiP (Fig 3B). Moreover, the number of peroxisome specific TiP proteins is significantly elevated in adrenal gland (nTiP = 26, *P*-value < 0.001, Fisher, Bonferroni), small intestine (nTiP = 22, *P*-value < 0.001, Fisher, Bonferroni), duodenum (nTiP = 28, *P*-value < 0.001, Fisher, Bonferroni), liver (nTiP = 84, *P*-value < 0.001, Fisher, Bonferroni), and kidney (nTiP = 45, *P*-value < 0.001, Fisher, Bonferroni) (Fig 3B).

To further examine what influences tissue-specificity of peroxisomal function and dysfunction, we followed the idea from Kitsak and coworkers that functional subnetworks of disease-associated proteins need to be expressed within a tissue for a disease to manifest (Kitsak et al, 2016). The identified ePI-disease module did vary slightly in size (n = 51–56) within the different tissues. Next, we asked whether we could find functional tissue-specific subnetworks of TiP. When calculating the lcc formed by peroxisomal TiP in each tissue, we found a more tissue-specific pattern mimicking the well-known clinical consequences of peroxisomal dysfunction. In 9 of the 50 tissues, the lcc of peroxisomal TiP is higher than expected by chance (Bonferroni-corrected empirical *P*-value < 0.05) (Fig 3D, Supplemental Data 6). Among them, tissues were well described as affected in peroxisomal disorders such as liver, adrenal gland, and the musculoskeletal system (midbrain and skeletal muscle) are represented. Again, the proteins that are most often represented in the tissue-specific lcc are mainly involved in metabolic functions of peroxisomes rather than in peroxisome biogenesis (Supplemental Data 7).

Supplemental Data 6. TiP modules per tissue.

Supplemental Data 7. Number of times a peroxisomal TiP protein is part of a TiP module.

To further characterize the biological relevance of the TiP modules, we assigned known protein functions to the respective modules (Supplemental Data 8). This analysis revealed tissue-specific differences. For example, in the brain tissue of the midbrain, the module is primarily associated with glyoxylate metabolism, reactive oxygen species, and amino-acid metabolism. In contrast, fatty acid oxidation is predominantly linked to metabolic tissues, including the liver, kidney, duodenum, and adipose tissue (Fig 3D).

Supplemental Data 8. Molecular functions of peroxisomal proteins.

### Tissue-specific PEX-TiP interactions point to mechanistic importance for peroxisomal function/dysfunction

Lastly, we considered the fact that peroxisomes are tightly connected to other organelles. Consequently, peroxisomal proteins need to interact with other non-peroxisomal proteins. To find potentially crucial peroxisomal to non-peroxisomal PPI, we searched for non-peroxisomal TiP that directly interact with one of the proteins from the tissue-specific ePIs. We find 1,272 different non-peroxisomal TiP interacting with at least one peroxisomal protein in at least one tissue (Supplemental Data 9). These non-peroxisomal TiPs have a higher degree and are more central within the human interactome, connecting the ePI to the cellular interactome (average degree 32.5 versus 7, *P*-value < 0.001; cc 0.00083 versus 0.00016, *P*-value < 0.001; bc 0.322 versus 0.289, *P*-value < 0.001; Mann–Whitney *U*). They are enriched in disease-associated proteins (odds 1.219, *P*-value = 0.002) and in drug targets (odds 1.217, *P*-value = 0.02) when compared to the HuInteractome, making them interesting targets for drug repurposing.

Supplemental Data 9. Non-pex TiP neighbors and their ePI interaction partners.

Interestingly, the proteolipid protein 1 (PLP1) is most often a TiP neighbor of the ePI (n = 11, Supplemental Data 9). Mutations in PLP1 result in failure of myelination and neurological dysfunction in the X-linked leukodystrophy Pelizaeus–Merzbacher disease (Inoue, 2005), like the peroxisomal disease X-ALD. There is no direct PPI between the causal protein of X-ALD, ABCD1, and PLP1, but studies showed hypermethylation of PLP1 in affected cells of X-ALD patients, and here, we provide a link to the protein layer of PLP1 and the peroxisomal interaction network (Manor et al, 2021).

**Figure 4.**
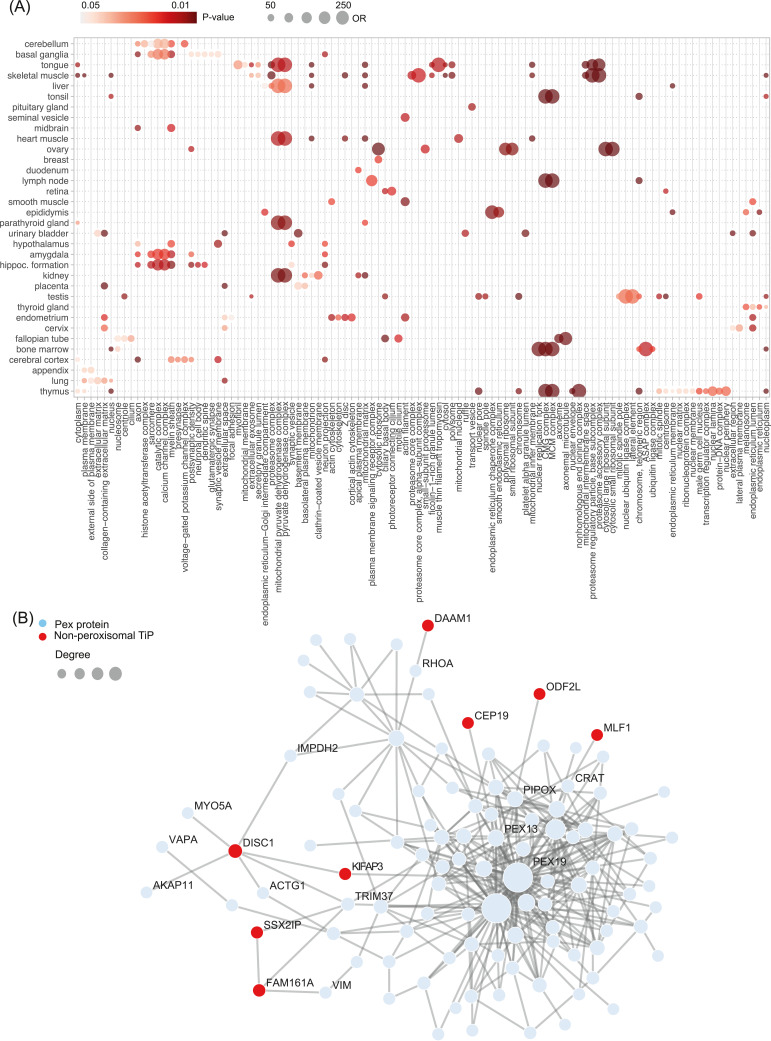
Tissue-specific peroxisomal-TiP interactions point to mechanistic importance for peroxisomal function. **(A)** Heatmap representation of enriched GO-CC terms per tissue from non-peroxisomal TiP neighbors. Only terms with a corrected *P*-value < 0.05 and more than two associated proteins are shown. The color represents the *P*-value, whereas the size corresponds to the calculated odds ratio (OR) of each term. The full list of enriched GO terms can be found in the Supplemental Data 10, 11, and 12. **(B)** Network representation of the retina-specific peroxisomal interactome and non-peroxisomal TiP neighbors (shown in red) associated with the enriched GO-CC terms “ciliary basal body” and “centrosome.” The size of a node reflects its degree; edges indicate known PPI between the corresponding proteins. The PPIs between the red nodes and peroxisomal proteins are of particular interest for further investigation into their mechanistic role in the pathogenesis of retinal dysfunction.

**Figure S5.**
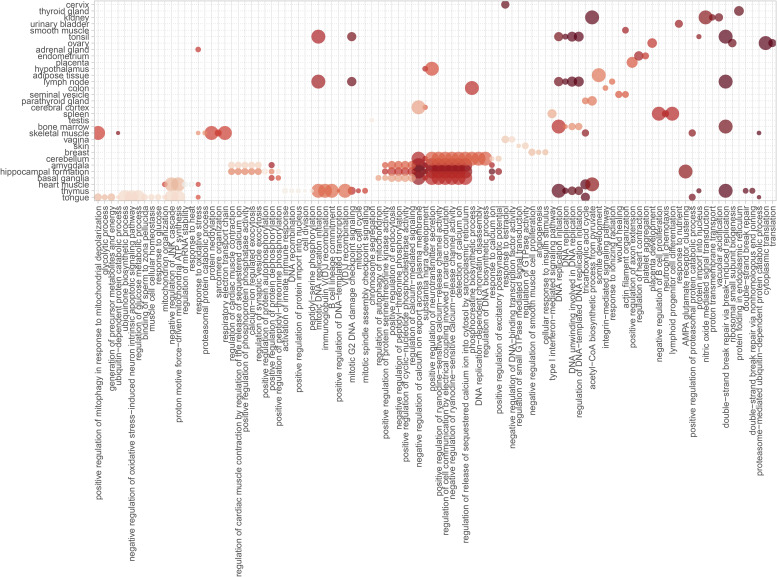
Heatmap representation of enriched GO-BP terms per tissue from non-peroxisomal TiP neighbors. Only terms with a corrected *P*-value < 0.05 and more than two associated proteins are shown. The color represents the *P*-value, whereas the size corresponds to the calculated odds ratio (OR) of each term.

**Figure S6.**
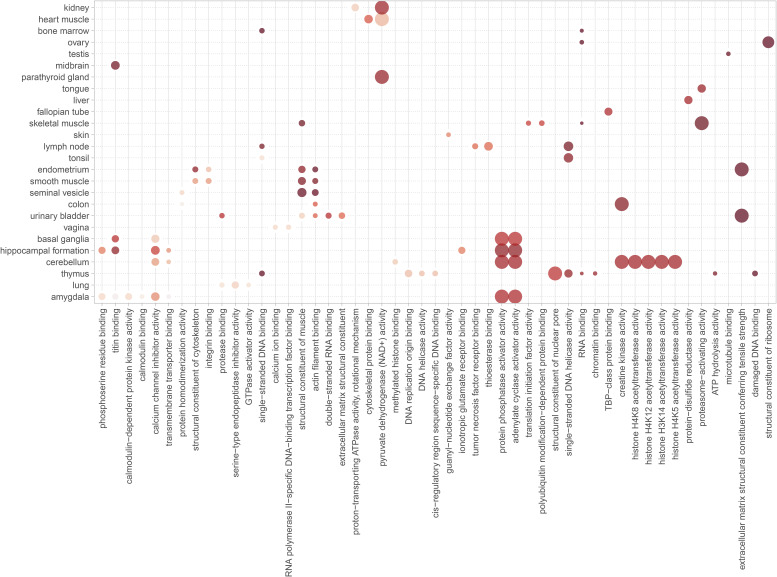
Heatmap representation of enriched GO-MF terms per tissue from non-peroxisomal TiP neighbors. Only terms with a corrected *P*-value < 0.05 and more than two associated proteins are shown. The color represents the *P*-value, whereas the size corresponds to the calculated odds ratio (OR) of each term.

To further investigate the biological functions of non-peroxisomal TiP neighbors, we performed Gene Ontology (GO) enrichment analyses for each set of TiP per tissue (Ashburner et al, 2000; Aleksander et al, 2026). This revealed distinctive enriched GO terms. For example, enriched cellular compartments were found in 34 of the 50 tissues (Figs 4A, S5, and S6, Supplemental Data 10, 11, and 12). We will outline how this information can be used to create new hypotheses on the underlying pathomechanisms of peroxisomal diseases.

Supplemental Data 10. GO enrichment of non-pex TiP neighbors for GO tag Biological Process (BP).

Supplemental Data 11. GO enrichment of non-pex TiP neighbors for GO tag Cellular Component (CC).

Supplemental Data 12. GO enrichment of non-pex TiP neighbors for GO tag Molecular Function (MF).

Retinopathy is a common symptom in peroxisomal disease, and the underlying cellular mechanisms are unclear (Kumar et al, 2024). In our retina-specific interactome, the direct non-peroxisomal TiP neighbors of the ePI are enriched for the GO terms “centrosome” (OR = 3.30, *P*-value = 0.01), “ciliary basal body” (OR = 7.55, *P*-value = 0.01), and “photoreceptor connecting cilium” (OR = 31.82, *P*-value = 0.02) (Fig 4A, Supplemental Data 11). The ciliary basal body plays a pivotal role in ciliogenesis, and its dysfunction may lead to so-called ciliopathies (Reiter & Leroux, 2017). One of the features of ciliopathies is retinal degradation. The phenotypic similarity with ciliopathies and the enrichment of proteins essential for ciliogenesis in the near proximity of the ePI point to a mechanistic importance of the underlying PPI (Fig 4B). Indeed, a possible link between peroxisomal dysfunction and ciliopathies has been hypothesized (Kumar et al, 2024), and here, we provide another link based on protein interactions between peroxisomal proteins and their direct non-peroxisomal neighbors (Fig 4B).

## Discussion

Although peroxisomal function and dysfunction have been extensively studied in recent years, many of the underlying cellular mechanisms remain poorly understood. Network-based approaches have become valuable tools for hypothesis generation, biomarker identification, and drug repurposing. However, the reliability of these approaches critically depends on the quality of the underlying experimental data—particularly the accuracy and completeness of PPI networks (Silverman et al, 2020).

To address this issue, our study (i) aimed to expand our understanding of peroxisomal PPIs and (ii) integrated the resulting data with network-based approaches to generate hypotheses about peroxisomal function and dysfunction. We screened 4,851 peroxisome-associated protein pairs and identified 403 PPIs including isoforms, and 333 PPIs without isoforms, thereby significantly broadening the existing peroxisomal interactome. The PPIs identified through our BRET-based screening method are of high quality, as recently demonstrated, primarily due to detection in living cells, which allows for the inclusion of post-translational modifications (Lotz-Havla et al, 2021a). An important aspect to consider is that the comparatively high number of newly identified peroxisomal PPIs may, at least in part, reflect the limited prior coverage of the peroxisomal interactome rather than an overestimation by our screening strategy. Nevertheless, BRET-based assays—particularly when applied to membrane-associated proteins in confined compartments—can be influenced by proximity-driven signals arising from local protein density or overexpression. Our assay conditions were empirically validated and carefully controlled (Lotz-Havla et al, 2021a), and based on our methodological assessment, we estimate that ∼10–12% of the 333 (without isoforms) newly identified interactions (corresponding to ∼40 candidates) may represent false-positive events. Despite these inherent limitations, the substantial expansion of the peroxisomal interaction network presented here provides a valuable framework for future hypothesis-driven and orthogonal validation studies. The systematic pairwise nature of our approach avoided the study bias commonly observed in disease-associated proteins (Luck et al, 2020). Specifically, in our screen, the average degree between non-disease and disease-associated proteins showed no significant difference. In contrast, the HuInteractome, which aggregates data from various interaction databases, displayed a significantly higher degree of disease-associated proteins, primarily due to their more frequent study. This again highlights the importance of conducting more unbiased interactome studies, such as the one presented here.

The mapped ePI represents a network area highly enriched in disease-associated proteins, drug targets, and homomers. The frequency of homomers in the ePI is consistent with a recent estimate that ∼30–50% of proteins form homomers (Levy & Teichmann, 2013). Moreover, due to the important metabolic functions of peroxisomes and the observation that homomerization is specifically important for metabolic enzymes (Frieden, 2019), this finding underscores the quality and biological relevance of the ePI.

Peroxins are crucial proteins required for peroxisome biogenesis and maintenance. Their dysfunction is associated with Zellweger spectrum disorders (Waterham et al, 2015). In the ePI, the biological significance of peroxins is emphasized by their higher average degree of connectivity compared to other peroxisomal proteins, highlighting their central role in peroxisomal network architecture. Our screen identified another hub protein, FIS1, which is dually localized to mitochondria and peroxisomes. Although its role in peroxisomal fission has only recently been established (Schrader et al, 2022), more specialized functions—such as a possible involvement in the development of Alzheimer’s Disease—have also been proposed (Ihenacho et al, 2021). The multiple PPIs we detected for FIS1 may reflect these additional, more specialized roles.

Network-based studies have led to the disease module hypothesis, that disease-associated proteins are not scattered randomly but tend to agglomerate in disease-specific regions (Menche et al, 2015). First, we found that the ePI is enriched in disease-associated proteins. Second, of the 89 disease-associated proteins in the ePI, 56 are directly connected with each other and form a disease module. These findings underscore the current view of peroxisomes as central players not only for metabolic functions but also for several other disorders such as cancer, viral infection, diabetes, and neurodegeneration (Zalckvar & Schuldiner, 2022). Because the ePI is also enriched in drug targets, the disease module can be further used for drug repurposing approaches, as recently proposed (Nogales et al, 2022). We found interesting candidates suitable for drug repurposing close to the peroxisomal disease module. Some of these candidates such as Taurine, Huperzine A, and Netarsudil, are currently under investigation for the treatment of Alzheimer’s Disease. This finding makes them not only promising treatment options in peroxisomal disease but also offers the possibility of new hypothesis generation about the underlying pathologic pathways of both diseases.

Peroxisomal function and dysfunction affect tissues to varying degrees. By integrating data on tissue-specific protein expression with our ePI, we aimed to investigate tissue-specific differences at the PPI level. First, we observed that most peroxisomal proteins are relatively uniformly expressed, leading us to conclude that expression alone is insufficient for generating new hypotheses on tissue-specific peroxisomal functions. Next, we built on the idea proposed by Luck and colleagues that tissue-specific functions are best explained by the presence of PPIs between TiPs and uniformly expressed proteins (Luck et al, 2020). Our analysis revealed that peroxisomal proteins are more frequently classified as TiPs when they are enzymes rather than peroxins. This aligns with recent findings that peroxisomes perform diverse tissue-specific functions, whereas essential processes such as peroxisomal biogenesis are not tissue-specific (Plessner et al, 2024). Additionally, the number of peroxisomal TiPs is significantly higher in tissues commonly affected by peroxisomal dysfunction, such as the liver, kidneys, and adrenal glands. Finally, within a network-based framework, tissue specificity can best be described by the presence of tissue-specific modules (Kitsak et al, 2016). Our analysis identified nine tissue-specific TiP modules, based on a pattern similar to the tissues affected in peroxisomal diseases. Functional analysis of these modules reflects known tissue-specific peculiarities. Further studies are needed to validate the biological significance of these modules. A recent study showed that >60% of all human disease-causing missense mutations lead to perturbations in PPI, a concept that is known as “edgetic perturbation” (Sahni et al, 2013). That said, mutations in peroxisomal disease-associated proteins may not only perturb metabolic pathways but also the network of their protein interactions. Based on GO enrichment analyses and the concept of non-peroxisomal TiP interactions, we identified several interesting PPIs that might play a mechanistic role in peroxisomal function and where edgetic perturbation could result in peroxisomal dysfunction. In particular, some of the identified interactions link peroxisomal proteins to pathways relevant for specialized cellular contexts, such as retinal or ciliary biology, thereby providing potential mechanistic connections between peroxisomal dysfunction and ciliopathies. The observed connections between retinal tissue-preferential proteins and components associated with the ciliary basal body or centrosome should be interpreted cautiously and primarily illustrate how the expanded peroxisomal interactome can be explored to generate hypotheses linking peroxisomal proteins to tissue-specific cellular pathways. Although targeted validation of selected interaction pairs, for example, by co-immunoprecipitation or colocalization analyses in appropriate cellular model systems, would provide additional biological insight, such experiments are beyond the scope of the present study. Instead, the dataset presented here provides a framework for future hypothesis-driven investigations aimed at experimentally validating and functionally characterizing these candidate interactions. For example, one could determine if a certain genetic variant in a peroxisomal protein leads to perturbation of an associated PPI, as our group has recently demonstrated for PEX26 (Lotz-Havla et al, 2021b).

Taken together, our integrative approach—combining experimental PPI mapping with computational network medicine—yields valuable insights into peroxisomal protein interactions and their mechanistic roles in peroxisomal function and dysfunction. The dataset presented here offers a robust foundation for hypothesis generation regarding the pathophysiology of peroxisomal disorders, supports drug repurposing strategies, and paves the way for further functional investigations. Moreover, our framework is readily adaptable for exploring interactome networks associated with other small organelles.

## Materials and Methods

### Selection of peroxisomal proteins and expanded peroxisomal interactome (ePI)

Peroxisomal proteins were selected with the help of previously published data (Yifrach et al, 2018). As some of the protein names in the dataset were outdated, we updated them to the official symbols (HRASLS—PLAAT1; PLA2G16—PLAAT3; SLC9A3R1—NHERF1; TMEM173—STING1; TMEM5—RXYLT1; ZADH2—PTGR3; MARC2—MTARC2; MARC1—MTARC1). During this process, HRSP12 and RIDA were merged into one protein. Of the 194 published peroxisomal genes, we identified 92 proteins in our in-house protein bank. 79% (73 out of 92) of the investigated proteins have a high peroxisomal detection rate (Yifrach et al, 2018). For six of those proteins, a second isoform could be investigated. A total of 98 proteins were screened for binary PPIs. To investigate the binding patterns of different isoforms these proteins were treated separately. However, to allow comparison with other databases (e.g., human interactome data, tissue expression data) the peroxisomal interactome (PI) was built without distinguishing between isoforms. In the case of the six proteins all interactions of both isoforms were joined. Finally, all proteins from the Yifrach dataset were translated to EntrezID (https://ftp.ncbi.nih.gov/gene/DATA/GENE_INFO/Mammalia/Homo_sapiens.gene_info.gz, downloaded 26 August 2023). ACAA1B, ECI3, ACAA1A, ACNAT, ACOT, ACNAT2, URAH, HSD3B3, ACOT5, SLC22A21, G3V3G9 being mouse proteins were not translated. This yielded 183 reference peroxisomal proteins. Of these, we found 161 to be present in the HuInteractome. This expands the 92 peroxisomal proteins in the PI to 173 proteins in the ePI.

### Binary protein interaction mapping by bioluminescence resonance energy transfer (iBRET)

#### Plasmids

For our automated informatics-aided iBRET process, Gateway destination vectors were designed based on the pcDNA 6.2 DEST general Gateway vector (Invitrogen). To generate BRET destination vectors with different tag orientations, the coding sequences of hRluc (phRG-TK) and Venus (pEYFP-N1) (Nagai et al, 2002), modified as previously described (Nagai et al, 2002; Hamdan et al, 2005), were amplified without a stop codon and fused 5′ in frame with the Gateway ccdB cassette to generate N-terminal fusion proteins, or amplified with a stop codon and fused 3′ in frame with the ccdB cassette to generate C-terminal fusion proteins.

Full-length ORFs with or without stop codons were obtained as Gateway Entry clones from the PlasmID database (Harvard Medical School PlasmID Repository), from the mammalian gene collection, or were amplified by single-step PCR from target open reading frames and introduced into appropriate Gateway Donor vectors. Sequences of all ORFs were verified by DNA sequence analysis (Eurofins MWG Operon).

BRET expression clones were generated by Gateway-based recombination of Entry clones without a stop codon with C-terminally fused hRluc or Venus destination vectors, or Entry clones with stop codon with N-terminally fused destination vectors, resulting in four expression vectors per protein of interest. A Venus–hRluc fusion construct was generated and used as a positive BRET control. Plasmids were propagated in *Escherichia coli* DH5α and purified using the PureYield plasmid maxiprep system (Promega).

#### Cell culture

HEK293 cells were maintained under standard monolayer culture conditions at 37°C and 5% CO_2_ in HYPERFlask cell culture vessels (Corning).

#### Automated iBRET process

DNA preparation, 96-well electroporation, incubation, and luminescence detection were fully integrated on an automated liquid-handling platform (Tecan Freedom EVO200). Transfections were performed in 96-well format using the Amaxa 96-well Shuttle system (Lonza), applying donor (hRluc) and acceptor (Venus) fusion constructs at an acceptor-to-donor ratio of 3:1 with a total of 0.6 μg DNA for all tag combinations. As a substrate, coelenterazine (PJK) was added to the living cells (30 μM), and emission was recorded at 475 nm (hRluc signal) and 535 nm (BRET signal) using a multimode reader enabling simultaneous dual emission (PHERAstar, BMG Labtech).

All liquid-handling steps were executed by independent 8- and 96-channel pipetting arms, and plates were transported by a robotic manipulator. After electroporation, cells were transferred to white 96-well plates (Corning) and incubated for 24 h at 37°C and 5% CO_2_.

An automated scheduling workflow implemented in EVOware software (Tecan) ensured uniform processing of all plates within defined runs. Raw data and processed results were stored in a MySQL database.

#### Experimental design

PPIs were analyzed in living cells by our automated iBRET process as described before and in the following sections(Lotz-Havla et al, 2021a). Because of the iBRET setup, eight different tag combinations per protein pair (or four tag combinations per homo-oligomerization) in duplicates were tested, resulting in a total of 38,416 BRET experiments.

For each tested protein pair, the respective BRET ratio per tag combination was calculated based on *R = I*_*A*_*/I*_*D*_*–cf*, where *R* is the BRET ratio, *I*_*A*_ is the intensity of light emission at 535 nm, *I*_*D*_ is the intensity of light emission at 475 nm, and *cf* is a correction factor (BRET_control_/Rluc_control_). To sort out transfection failures and reduce false-positive results, BRET ratios were not calculated if *I*_*D*_ was below a pre-calculated cutoff value, which was 18,358.5 for all screened protein pairs, despite protein pairs from the PEX11 screen (PEX11A, PEX11B, or PEX11G against all other included proteins), where the cutoff was 6,822. A protein pair was considered interacting if at least one out of eight tested combinations resulted in a BRET ratio above a predefined threshold, which was 0.0263 and 0.0284 for the PEX11 screen. The *I*_*D*_ cutoffs as well as the thresholds were calculated applying a reference data set of known interacting and randomly chosen—potentially non-interacting-protein pairs (for details see below and as recently described (Lotz-Havla et al, 2021a).

#### Curation of reference data sets (positive reference set [PRS] and random reference set [RRS])

A PRS comprising 61 documented protein–protein interactions was curated from PubMed and interaction databases including HPRD, STRING, BIND, and BioGRID (Bader et al, 2003; Prasad et al, 2009; Oughtred et al, 2021; Szklarczyk et al, 2023). All interactions were manually verified and assigned confidence scores based on the number of independent experimental confirmations.

An RRS consisting of 60 protein pairs was generated by random sampling from an in-house cDNA library. Protein pairs were excluded if interactions were reported in databases or if both proteins shared similar GO cellular component annotations. This procedure was repeated until 60 valid random pairs were obtained. We provide the PRS and RRS in Zenodo (see below).

### Data processing and analysis

We used Python for data processing and analysis (Python Software Foundation, version 3.9.4 available at https://www.python.org/) on macOS Big Sur Version 11.7. More specifically, we used mainly the following packages: pandas 1.2.4, numpy 1.20.2, scipy 1.6.3, goatools 1.1.6, networkx 2.5.1. Visualization and statistical tests (if not already done in Python) have been done using R (R version 4.4.1 https://www.r-project.org) and mainly the package tidyverse 2.0.0.

#### Analysis of degree distributions

Degree distributions were analyzed using the poweRlaw package (Version 1.00). Discrete power-law models were fitted and evaluated using bootstrap-based Kolmogorov–Smirnov goodness-of-fit tests (function bootstrap_p, 1,000 sims). Model comparison against a log–normal distribution was performed using likelihood-ratio testing following the approach of Clauset (Clauset et al, 2009). All analyses were restricted to the distribution tail above the estimated xmin.

### Calculation of network parameters

Basic graph parameters such as degree, diameter, density, closeness centrality, and betweenness centrality were calculated using the package NetworkX (NetworkX Developers, version 2.5.1). Closeness (cc) and betweenness centrality (bc) are centrality measures where the first is defined as the inverse average of all shortest paths from a node to all other reachable nodes in the network and thereby quantifies how close a node is to all others in the network. The latter is a measure of centrality in a network that quantifies how often a node lies on the shortest paths between other nodes. To address the power-law distribution of the network, all average degrees are defined as the median of the degrees of a given group of nodes (numpy, median).

Although basic parameters were calculated for all screened proteins (including the isoforms; n = 98), later comparative analysis was performed using only the main proteins (n = 92).

### Jaccard index

Jaccard Index was calculated as follows:

$$J\left(proteinA,proteinB\right)=\left(\frac{proteinA\;\cap \;proteinB}{proteinA\;\cup \;proteinB}\right)$$

In this equation, proteinA and proteinB represent all interactions of two different peroxisomal proteins A and B. A higher index indicates a greater overlap of the interactions of the two proteins.

### Statistics

We used Kruskal–Wallis (scipy.stats, kruskal), Wilcoxon-rank test (R ggpubr), Mann– Whitney *U* (scipy.stats, mannwhitneyu), Fold enrichment (scipy.stats, fisher_exact). All other statistical tests are mentioned in the respective sections.

### Human Interactome

**Figure S7.**
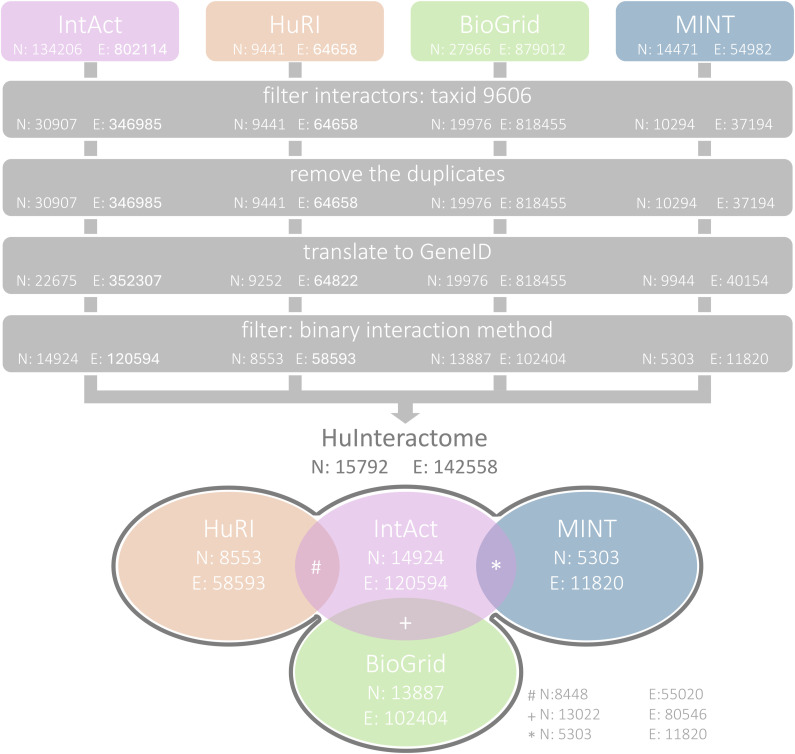
Flowchart presenting the data processing steps applied to generate the HuInteractome. The databases IntAct, HuRI, BioGrid, and MINT were separately filtered to contain only interactions where both interactors are tagged with taxid 9606, followed by removing duplicated entries. Then, the protein names were translated to GeneID: if one protein name mapped to several GeneIDs, all GeneIDs were added; if no translation was found, the entry was deleted. In the last step, we filtered the resulting interactions for binary interaction methods. N = nodes and E = edges of a graph containing all entries of the current processing step.

To investigate the role of the peroxisomal interactome in the context of the entire human interactome, we compiled interaction data from four sources (BioGrid, HuRI [Hi-union.psi], MINT, IntAct, downloaded 26 August 2023) (Licata et al, 2012; Orchard et al, 2014; Luck et al, 2020). First, we processed the data to contain only PPIs between human proteins (taxid: 9606). Then, if applicable, protein names were translated to Entrez ID using UniProt Database (https://ftp.uniprot.org/pub/databases/uniprot/previous_major_releases/release-2023_03/knowledgebase/uniprot_sprot-only2023_03.tar.gz, downloaded 26 August 2023) (Bateman et al, 2025), and if not successful, HGNC database (https://www.genenames.org/download/archive/monthly/tsv/, downloaded 26 August 2023) (Seal et al, 2026). PPIs where one partner could not be translated were excluded; if multiple translations were found, all translations were considered. Finally, interactions were filtered, which were detected by a binary interaction detection method as published by Alonso-Lopez (Alonso-López et al, 2019). A flowchart depicting all the filtering steps for each interactome dataset can be found in the supplementary material (Fig S7). The resulting interactome contains 15,792 proteins and 142,558 interactions and will be referred to as Human Interactome (HuInteractome, Supplemental Data 3).

### Network randomization

To account for inherent dependencies within protein interaction networks, several analyses were evaluated relative to randomized background networks that preserve key topological properties, particularly node degree. Degree-preserving randomization was used to generate null distributions against which observed network properties and proximity scores were compared. For further details, please see each section.

### Disease-associated proteins and creation of the peroxisomal disease module

To build the disease module, we extracted all peroxisomal proteins listed in the human phenotype ontology (HPO) (https://github.com/obophenotype/human-phenotype-ontology/releases/download/v2023-09-01/genes_to_phenotype.txt, downloaded 2 September 2023) (Gargano et al, 2024). We considered each peroxisomal protein associated with an OMIM number as a disease-associated protein, and proteins not associated as non-disease-associated proteins, respectively. The lcc of all peroxisomal disease-associated proteins was considered to be the peroxisomal disease module (ePI-disease module). To determine whether the peroxisomal disease module was greater than expected, we generated 1000-degree matched samples using the function gen_degree_preserved_sets as published by Maiorino et al (2020) from the entire HuInteractome, defined the lcc, and calculated a z-score and an empirical *P*-value accordingly.

$$z-score=\frac{observedLCC-mean\left(randomLCC\right)}{std\left(randomLCC\right)}$$

$$p_{emp}=\frac{sum\left(randomLCC\geq observedLCC\right)+1}{1000+1}$$

Where *observedLCC* presents the size of the lcc of the peroxisomal disease genes and *randomLCC* the sizes of the lcc of the proteins acquired by 1,000-degree matched random drawings.

### Drug targets and drug repurposing

To identify drugs possibly interacting with the peroxisomal disease-associated proteins and with the potential to be used for treatment, we chose all approved but not withdrawn drugs from the DrugBank database (https://go.drugbank.com/releases/, version 5.1.10, downloaded 2 September 2023) (Knox et al, 2024). We obtained 2,186 different drugs and calculated the mean minimal distance for all drug targets of a given drug to the ePI-disease module. Secondly, we draw 1,000-degree matched (Maiorino et al, 2020) samples of drug targets and disease modules for each drug and calculated the respective z-score.

$$z-score=\frac{\left(observedD-mean\left(randomD\right)\right)}{std\left(randomD\right)}$$

Where *observedD* is the mean closest distance of all drug targets of a drug to the disease module, and *randomD* represents the mean closest distance of the 1,000-degree matched samples. Network proximity was primarily used as an exploratory measure to prioritize candidate compounds. Accordingly, results were interpreted in terms of relative rankings and deviations from randomized network expectations rather than as independent hypothesis tests across all comparisons.

### Integration of human protein atlas data

To investigate tissue specificity of the peroxisomal proteins we used the Human Protein Atlas (HPA) (https://v23.proteinatlas.org/download/rna_tissue_consensus.tsv.zip, downloaded 26 August 2023, the data is based on the HPA version 23.0 and Ensembl version 109) (Uhlen et al, 2010). Briefly, the HPA offers integrated transcript expression levels per gene for 50 different tissues. Ensembl identifiers have been mapped to Entrez ID using HGNC and NCBI (https://ftp.ncbi.nih.gov/gene/DATA/GENE_INFO/Mammalia/Homo_sapiens.gene_info.gz) database (both downloaded 26 August 2023) (Sayers et al, 2025). 355 Ensembl IDs could not be translated. 172 of 173 peroxisomal proteins were present in the HPA. A protein was considered expressed in the respective tissue if its expression level was measured above 1.0. Tissue-specific ePI only contained all expressed proteins of a given tissue. In line with the previously published analysis (Luck et al, 2020), we determined tissue-preferentially expressed proteins (TiP). Therefore, the expression level was normalized for each protein across all tissues where it was considered to be expressed. Normalization was performed using the RobustScaler from scikit-learn (scikit-learn developers, version 1.0.1). A TiP was finally determined with an absolute expression level above 5.0 and a normalized expression level above 2.0.

### Functional analysis of peroxisomal TiP proteins

To differentiate the peroxisomal function in different tissues we identified the TiP peroxisomal proteins of each tissue. We picked only the TiP forming the largest subgraph (lcc of the peroxisomal TiP proteins). Functions of TiP were manually curated from the peroxisomedb (http://www.peroxisomedb.org) (Schlüter et al, 2009) and the Yifrach dataset (Yifrach et al, 2018) (Supplemental Data 8).

### GO enrichment of TiP neighbors

To investigate the functions of non-peroxisomal TiP neighbors, GO enrichment analysis was performed. GO terms were retrieved from https://release.geneontology.org/2023-07-27/ontology/go-basic, (data release 2023-07-27) and http://ftp.ncbi.nlm.nih.gov/gene/DATA/gene2go.gz (downloaded 26 August September 2023). For reading the ontology we used the goatools READER (Klopfenstein et al, 2018). Enrichment was calculated using the fisher_exact function of the SciPy package (version 1.6.3). Background was defined as all direct neighbors of the ePI which are part of the HPA database. We used alpha = 0.05 and corrected with fdrcorrection from statsmodels (version 0.12.2). A non-peroxisomal TiP neighbor in a tissue was defined as being both a TiP in the tissue and a direct neighbor of the tissue-specific peroxisomal lcc but not a peroxisomal protein.

### Random comparison of tissue-specific peroxisomal largest-connected components (lcc) and lcc of tissue-preferentially expressed proteins (TiP)

We tested whether the lcc build by tissue-specific peroxisomal proteins and the peroxisomal TiP were, respectively, larger than chance. Therefore, we randomly drew 1,000-degree matched samples from the HUInteratome. We calculated the z-score and empirical *P*-value as stated above. *P*-values were Bonferroni corrected.

## Data and Code Availability

The data and code supporting the findings of this study are publicly available. All in-house datasets, as well as the public databases we used that are not archived, can be found in Zenodo (Gersting et al, 2026). Additional raw data is available from the corresponding author on reasonable request. The Python code used for data processing and analysis is available at https://github.com/julavcramer/PEX, the R scripts used for complementary analysis and visualization are provided at https://github.com/mwoidy/pexInteractomeR.

The peroxisomal interaction dataset has been submitted to Biogrid and is available via https://wiki.thebiogrid.org/doku.php/gersting2026.

Further we used the following publicly available datasets:

• Protein expression data: HPA version 23 https://v23.proteinatlas.org/download/rna_tissue_consensus.tsv.zip
• Protein annotation: Gene Ontology, http://geneontology.org/ontology/go-basic.obohttp://geneontology.org/ontology/go-basic.obo data release 2023-07-27; https://release.geneontology.org/2023-07-27/ontology/go-basic.obo
• Protein names: Uniprot database, https://ftp.uniprot.org/pub/databases/uniprot/previous_major_releases/release-2023_03/knowledgebase/uniprot_sprot-only2023_03.tar.gz
• Protein names: HGNC database, https://www.genenames.org/download/archive/monthly/tsv/ https://storage.googleapis.com/public-download-files/hgnc/archive/archive/monthly/tsv/; as HGNC does not store the “gene_with_protein_product.txt” files we used, the closest is the “hgnc_complete_set.txt” release 2023-09-01 filtered for “locus_group” = “protein-coding gene” AND “status” = approved. However, the result differs slightly from the originally downloaded view of the data. Hence, we provide the datasets–as downloaded–in Zenodo
• Protein names: NCBI database, https://ftp.ncbi.nih.gov/gene/DATA/GENE_INFO/Mammalia/Homo_sapiens.gene_info.gz; however NCBI does not provide archived data so the dataview used in this project is provided in Zenodo
• Disease annotation: Human phenotype ontology, https://github.com/obophenotype/human-phenotype-ontology/releases/download/v2023-09-01/genes_to_phenotype.txt
• Drug annotation: Drugbank, https://go.drugbank.com/releases/, version 5.1.10,
• Peroxisomal proteins: Yifrach et al (2018)
• Peroxisomal protein annotation: Peroxisome Database, http://www.peroxisomedb.org

For compiling the interactome:

• Biogrid: https://downloads.thebiogrid.org/Download/BioGRID/Release-Archive/BIOGRID-4.4.225/BIOGRID-ORGANISM-4.4.225.mitab.zip, file BIOGRID-ORGANISM-Homo_sapiens-4.4.225.mitab.txt
• Mint: retrieved via PSICQUIC (Aranda et al, 2011) on 26 August 2023 http://www.ebi.ac.uk/Tools/webservices/psicquic/mint/webservices/current/search/query/taxidA:9606?taxidB:9606 reflecting the intact database release 2023-06-02
• Intact: retrieved via PSICQUIC on 26 August 2023, http://www.ebi.ac.uk/Tools/webservices/psicquic/intact/webservices/current/search/query/taxidA:9606?taxidB:9606; reflecting the intact database release 2023-06-02
• Huri: http://www.interactome-atlas.org/data/HI-union.psi (Luck et al, 2020)
• In-house compiled interactome: HuInteractome, Supplemental Data 3 and provided in Zenodo

## Supplementary Material

Reviewer comments

## References

[bib1] Aleksander SA, Balhoff JP, Carbon S, Cherry JM, Ebert D, Feuermann M, Gaudet P, Harris NL, Hill DP, Kalita P, (2026) The gene ontology knowledgebase in 2026. Nucleic Acids Res 54: D1779–D1792. 10.1093/nar/gkaf1292 41413728 PMC12807639

[bib2] Alonso-López D, Campos-Laborie FJ, Gutiérrez MA, Lambourne L, Calderwood MA, Vidal M, De Las Rivas J (2019) APID database: redefining protein-protein interaction experimental evidences and binary interactomes. Database (Oxford) 2019: baz005. 10.1093/database/baz005 30715274 PMC6354026

[bib3] Antonicka H, Lin ZY, Janer A, Aaltonen MJ, Weraarpachai W, Gingras AC, Shoubridge EA (2020) A high-density human mitochondrial proximity interaction network. Cell Metab 32: 479–497.e9. 10.1016/j.cmet.2020.07.017 32877691

[bib4] Aranda B, Blankenburg H, Kerrien S, Brinkman FSL, Ceol A, Chautard E, Dana JM, De Las Rivas J, Dumousseau M, Galeota E, (2011) PSICQUIC and PSISCORE: Accessing and scoring molecular interactions. Nat Methods 8: 527–528. 10.1038/nmeth.1627 21716279 PMC3246345

[bib5] Ashburner M, Ball CA, Blake JA, Botstein D, Butler H, Cherry JM, Davis AP, Dolinski K, Dwight SS, Eppig JT, (2000) Gene ontology: tool for the unification of biology. The Gene Ontology Consortium. Nat Genet 25: 25–29. 10.1038/75556 10802651 PMC3037419

[bib6] Bader GD, Betel D, Hogue CWV (2003) BIND: The Biomolecular interaction network database. Nucleic Acids Res 31: 248–250. 10.1093/nar/gkg056 12519993 PMC165503

[bib7] Baliou S, Adamaki M, Ioannou P, Pappa A, Panayiotidis MI, Spandidos DA, Christodoulou I, Kyriakopoulos AM, Zoumpourlis V (2021) Protective role of taurine against oxidative stress. Mol Med Rep 24: 605. 10.3892/mmr.2021.12242 34184084 PMC8240184

[bib8] Barabási A-L, Gulbahce N, Loscalzo J (2011) Network medicine: A network-based approach to human disease. Nat Rev Genet 12: 56–68. 10.1038/nrg2918 21164525 PMC3140052

[bib9] Bateman A, Martin MJ, Orchard S, Magrane M, Adesina A, Ahmad S, Bowler-Barnett EH, Bye-A-Jee H, Carpentier D, Denny P, (2025) UniProt: The Universal protein knowledgebase in 2025. Nucleic Acids Res 53: D609–D617. 10.1093/nar/gkae1010 39552041 PMC11701636

[bib10] Bis-Brewer DM, Danzi MC, Wuchty S, Züchner S (2019) A network biology approach to unraveling inherited axonopathies. Sci Rep 9: 1–12. 10.1038/s41598-018-37119-z 30737464 PMC6368620

[bib11] Boldt K, Van Reeuwijk J, Lu Q, Koutroumpas K, Nguyen TMT, Texier Y, Van Beersum SEC, Horn N, Willer JR, Mans DA, (2016) An organelle-specific protein landscape identifies novel diseases and molecular mechanisms. Nat Commun 7: 1–13. 10.1038/ncomms11491 PMC4869170 27173435

[bib12] Braun F, Abed A, Sellung D, Rogg M, Woidy M, Eikrem O, Wanner N, Gambardella J, Laufer SD, Haas F, (2023) Synuclein alpha accumulation mediates podocyte injury in Fabry nephropathy. J Clin Invest 133: e157782. 10.1172/JCI157782 37014703 PMC10232004

[bib13] Caldera M, Buphamalai P, Müller F, Menche J (2017) Interactome-based approaches to human disease. Curr Opin Syst Biol 3: 88–94. 10.1016/j.coisb.2017.04.015

[bib14] Carmichael RE, Islinger M, Schrader M (2022) Fission Impossible (?)—new insights into disorders of peroxisome Dynamics. Cells 11: 1922. 10.3390/cells11121922 35741050 PMC9221819

[bib15] Cheng F, Kovács IA, Barabási A-L (2019) Network-based prediction of drug combinations. Nat Commun 10: 1197. 10.1038/s41467-019-09186-x 30867426 PMC6416394

[bib16] Clauset A, Shalizi CR, Newman MEJ (2009) Power-law distributions in empirical data. SIAM Rev 51: 661–703. 10.1137/070710111

[bib17] Comstra HS, McArthy J, Rudin-Rush S, Hartwig C, Gokhale A, Zlatic SA, Blackburn JB, Werner E, Petris M, D’Souza P, (2017) The interactome of the copper transporter ATP7A belongs to a network of neurodevelopmental and neurodegeneration factors. Elife 6: e24722. 10.7554/eLife.24722.001 28355134 PMC5400511

[bib18] Engelen M, Kemp S, De Visser M, Van Geel BM, Wanders RJA, Aubourg P, Poll-The BT (2012) X-linked adrenoleukodystrophy (X-ALD): Clinical presentation and guidelines for diagnosis, follow-up and management. Orphanet J Rare Dis 7: 51. 10.1186/1750-1172-7-51 22889154 PMC3503704

[bib19] Frieden C (2019) Protein oligomerization as a metabolic control mechanism: Application to apoE. Protein Sci 28: 837–842. 10.1002/pro.3583 30701627 PMC6423707

[bib20] Friedli MJ, Inestrosa NC (2021) Huperzine a and its neuroprotective molecular signaling in Alzheimer’s disease. Molecules 26: 6531. 10.3390/molecules26216531 34770940 PMC8587556

[bib21] Galletta BJ, Fagerstrom CJ, Schoborg TA, McLamarrah TA, Ryniawec JM, Buster DW, Slep KC, Rogers GC, Rusan NM (2016) A centrosome interactome provides insight into organelle assembly and reveals a non-duplication role for Plk4. Nat Commun 7: 12476. 10.1038/ncomms12476 27558293 PMC5007297

[bib22] Gargano MA, Matentzoglu N, Coleman B, Addo-Lartey EB, Anagnostopoulos AV, Anderton J, Avillach P, Bagley AM, Bakštein E, Balhoff JP, (2024) The human phenotype ontology in 2024: Phenotypes around the world. Nucleic Acids Res 52: D1333–D1346. 10.1093/nar/gkad1005 37953324 PMC10767975

[bib23] Gersting S, Cramer JV, Guder P, Lotz-Havla A, Wolf B, Noll-Puchta H, Erdmann R, Muntau AC, Woidy M (2026) Data for “Peroxisomal interactome mapping enables network-based modelling of function and disease.” by Gersting et al. Zenodo. 10.5281/zenodo.20443272 PMC13324248 42386525

[bib24] Guney E, Menche J, Vidal M, Barábasi AL (2016) Network-based in silico drug efficacy screening. Nat Commun 7: 10331. 10.1038/ncomms10331 26831545 PMC4740350

[bib25] Hamdan FF, Audet M, Garneau P, Pelletier J, Bouvier M (2005) High-throughput screening of G protein-coupled receptor antagonists using a bioluminescence resonance energy transfer 1-based beta-arrestin2 recruitment assay. J Biomol Screen 10: 463–475. 10.1177/1087057105275344 16093556

[bib26] Hashimoto K, Panchenko AR (2010) Mechanisms of protein oligomerization, the critical role of insertions and deletions in maintaining different oligomeric states. Proc Natl Acad Sci U S A 107: 20352–20357. 10.1073/pnas.1012999107 21048085 PMC2996646

[bib27] Huttlin EL, Bruckner RJ, Navarrete-Perea J, Cannon JR, Baltier K, Gebreab F, Gygi MP, Thornock A, Zarraga G, Tam S, (2021) Dual proteome-scale networks reveal cell-specific remodeling of the human interactome. Cell 184: 3022–3040.e28. 10.1016/j.cell.2021.04.011 33961781 PMC8165030

[bib28] Ihenacho UK, Meacham KA, Harwig MC, Widlansky ME, Hill RB (2021) Mitochondrial fission protein 1: Emerging roles in organellar form and function in health and disease. Front Endocrinol (Lausanne) 12: 660095. 10.3389/FENDO.2021.660095/FULL 33841340 PMC8027123

[bib29] Inoue K (2005) PLP1-related inherited dysmyelinating disorders: Pelizaeus-Merzbacher disease and spastic paraplegia type 2. Neurogenetics 6: 1–16. 10.1007/s10048-004-0207-y 15627202

[bib30] Jangra A, Gola P, Singh J, Gond P, Ghosh S, Rachamalla M, Dey A, Iqbal D, Kamal M, Sachdeva P, (2024) Emergence of taurine as a therapeutic agent for neurological disorders. Neural Regen Res 19: 62–68. 10.4103/1673-5374.374139 37488845 PMC10479846

[bib31] Kemp S, Huffnagel IC, Linthorst GE, Wanders RJ, Engelen M (2016) Adrenoleukodystrophy – neuroendocrine pathogenesis and redefinition of natural history. Nat Rev Endocrinol 12: 606–615. 10.1038/nrendo.2016.90 27312864

[bib32] Kitsak M, Sharma A, Menche J, Guney E, Ghiassian SD, Loscalzo J, Barabási AL (2016) Tissue specificity of human disease module. Nat Sci Rep 6: 35241. 10.1038/srep35241 PMC5066219 27748412

[bib33] Klopfenstein DV, Zhang L, Pedersen BS, Ramírez F, Vesztrocy AW, Naldi A, Mungall CJ, Yunes JM, Botvinnik O, Weigel M, (2018) GOATOOLS: A Python library for gene ontology analyses. Sci Rep 8: 10872. 10.1038/s41598-018-28948-z 30022098 PMC6052049

[bib34] Klouwer FCC, Berendse K, Ferdinandusse S, Wanders RJA, Engelen M, Poll-The BT (2015) Zellweger spectrum disorders: Clinical overview and management approach Inherited metabolic diseases. Orphanet J Rare Dis 10: 151. 10.1186/s13023-015-0368-9 26627182 PMC4666198

[bib35] Knox C, Wilson M, Klinger CM, Franklin M, Oler E, Wilson A, Pon A, Cox J, Chin NEL, Strawbridge SA, (2024) DrugBank 6.0: The DrugBank knowledgebase for 2024. Nucleic Acids Res 52: D1265–D1275. 10.1093/nar/gkad976 37953279 PMC10767804

[bib73] Kumar R, Islinger M, Worthy H, Carmichael R, Schrader M (2024) The peroxisome: An update on mysteries 3.0. Histochem Cell Biol 161: 99–132. 10.1007/s00418-023-02259-5 38244103 PMC10822820

[bib37] Levy ED, Teichmann S (2013) Structural, Evolutionary, and assembly principles of protein oligomerization. Prog Mol Biol Transl Sci 117: 25–51. 10.1016/B978-0-12-386931-9.00002-7 23663964

[bib38] Licata L, Briganti L, Peluso D, Perfetto L, Iannuccelli M, Galeota E, Sacco F, Palma A, Nardozza AP, Santonico E, (2012) MINT, the molecular interaction database: 2012 update. Nucleic Acids Res 40: 857–861. 10.1093/nar/gkr930 PMC3244991 22096227

[bib39] Lotz-Havla AS, Woidy M, Guder P, Friedel CC, Klingbeil JM, Bulau A-M, Schultze A, Dahmen I, Noll-Puchta H, Kemp S, (2021a) iBRET screen of the ABCD1 peroxisomal network and Mutation-Induced network perturbations. J Proteome Res 20: 4366–4380. 10.1021/acs.jproteome.1c00330 34383492

[bib40] Lotz-Havla AS, Woidy M, Guder P, Schmiesing J, Erdmann R, Waterham HR, Muntau AC, Gersting SW (2021b) Edgetic perturbations contribute to phenotypic Variability in PEX26 Deficiency. Front Genet 12: 726174. 10.3389/fgene.2021.726174 34804114 PMC8600046

[bib41] Luck K, Kim DK, Lambourne L, Spirohn K, Begg BE, Bian W, Brignall R, Cafarelli T, Campos-Laborie FJ, Charloteaux B, (2020) A reference map of the human binary protein interactome. Nature 580: 402–408. 10.1038/s41586-020-2188-x 32296183 PMC7169983

[bib42] Maiorino E, Baek SH, Guo F, Zhou X, Kothari PH, Silverman EK, Barabási A, Weiss ST, Raby BA, Sharma A (2020) Discovering the genes mediating the interactions between chronic respiratory diseases in the human interactome. Nat Commun 11: 811. 10.1038/s41467-020-14600-w 32041952 PMC7010776

[bib43] Manor J, Chung H, Bhagwat PK, Wangler MF (2021) ABCD1 and X-linked adrenoleukodystrophy: A disease with a markedly variable phenotype showing conserved neurobiology in animal models. J Neurosci Res 99: 3170–3181. 10.1002/jnr.24953 34716609 PMC9665428

[bib44] Menche J, Sharma A, Kitsak M, Ghiassian SD, Vidal M, Loscalzo J, Barabási A-L (2015) Disease networks. Uncovering disease-disease relationships through the incomplete interactome. Science 347: 1257601. 10.1126/science.1257601 25700523 PMC4435741

[bib45] Misselbeck K, Parolo S, Lorenzini F, Savoca V, Leonardelli L, Bora P, Morine MJ, Mione MC, Domenici E, Priami C (2019) A network-based approach to identify deregulated pathways and drug effects in metabolic syndrome. Nat Commun 10: 5215. 10.1038/s41467-019-13208-z 31740673 PMC6861239

[bib46] Nagai T, Ibata K, Park ES, Kubota M, Mikoshiba K, Miyawaki A (2002) A variant of yellow fluorescent protein with fast and efficient maturation for cell-biological applications. Nat Biotechnol 20: 87–90. 10.1038/nbt0102-87 11753368

[bib47] Nogales C, Mamdouh ZM, List M, Kiel C, Casas AI, Schmidt HHHW (2022) Network pharmacology: Curing causal mechanisms instead of treating symptoms. Trends Pharmacol Sci 43: 136–150. 10.1016/j.tips.2021.11.004 34895945

[bib48] Orchard S, Ammari M, Aranda B, Breuza L, Briganti L, Broackes-Carter F, Campbell NH, Chavali G, Chen C, Del-Toro N, (2014) The MIntAct project - IntAct as a common curation platform for 11 molecular interaction databases. Nucleic Acids Res 42: 358–363. 10.1093/nar/gkt1115 PMC3965093 24234451

[bib49] Oughtred R, Rust J, Chang C, Breitkreutz BJ, Stark C, Willems A, Boucher L, Leung G, Kolas N, Zhang F, (2021) The BioGRID database: A comprehensive biomedical resource of curated protein, genetic, and chemical interactions. Protein Sci 30: 187–200. 10.1002/pro.3978 33070389 PMC7737760

[bib50] Plessner M, Thiele L, Hofhuis J, Thoms S (2024) Tissue-specific roles of peroxisomes revealed by expression meta-analysis. Biol Direct 19: 14. 10.1186/s13062-024-00458-1 38365851 PMC10873952

[bib51] Prasad KTS, Goel R, Kandasamy K, Keerthikumar S, Kumar S, Mathivanan S, Telikicherla D, Raju R, Shafreen B, Venugopal A, (2009) Human protein reference database - 2009 update. Nucleic Acids Res 37: 767–772. 10.1093/nar/gkn892 PMC2686490 18988627

[bib52] Reiter JF, Leroux MR (2017) Genes and molecular pathways underpinning ciliopathies. Nat Rev Mol Cell Biol 18: 533–547. 10.1038/nrm.2017.60 28698599 PMC5851292

[bib53] Sahni N, Yi S, Zhong Q, Jailkhani N, Charloteaux B, Cusick ME, Vidal M (2013) Edgotype: A fundamental link between genotype and phenotype. Curr Opin Genet Dev 23: 649–657. 10.1016/j.gde.2013.11.002 24287335 PMC3902775

[bib54] Sayers EW, Beck J, Bolton EE, Brister JR, Chan J, Connor R, Feldgarden M, Fine AM, Funk K, Hoffman J, (2025) Database resources of the National center for Biotechnology information in 2025. Nucleic Acids Res 53: D20–D29. 10.1093/nar/gkae979 39526373 PMC11701734

[bib55] Schlüter A, Real-Chicharro A, Gabaldón T, Sánchez-Jiménez F, Pujol A (2009) PeroxisomeDB 2.0: An integrative view of the global peroxisomal metabolome. Nucleic Acids Res 38: D800–D805. 10.1093/nar/gkp935 19892824 PMC2808949

[bib56] Schrader M, Fahimi HD (2006) Peroxisomes and oxidative stress. Biochim Biophys Acta Mol Cell Res 1763: 1755–1766. 10.1016/j.bbamcr.2006.09.006 17034877

[bib57] Schrader TA, Carmichael RE, Islinger M, Costello JL, Hacker C, Bonekamp NA, Weishaupt JH, Andersen PM, Schrader M (2022) PEX11β and FIS1 cooperate in peroxisome division independent of Mitochondrial Fission Factor. J Cell Sci 135: jcs259924. 10.1242/jcs.259924 35678336 PMC9377713

[bib58] Seal RL, Braschi B, Gray K, McClay J, Tweedie S, Bruford EA (2026) Genenames.org: The HGNC and PGNC resources in 2026. Nucleic Acids Res 54: D1098–D1107. 10.1093/nar/gkaf1229 41287213 PMC12807706

[bib59] Sharma A, Kitsak M, Cho MC, Ameli A, Zhou X, Jiang Z, Crapo JD, Beaty TH, Menche J, Bakke PS, (2018) Integration of molecular interactome and targeted interaction analysis to identify a COPD disease network module. Sci Rep 8: 408229. 10.1101/408229 PMC6160419 30262855

[bib60] Silverman EK, Schmidt HHHW, Anastasiadou E, Altucci L, Angelini M, Badimon L, Balligand JL, Benincasa G, Capasso G, Conte F, (2020) Molecular networks in Network Medicine: Development and applications. Wiley Interdiscip Rev Syst Biol Med 12: e1489. 10.1002/wsbm.1489 32307915 PMC7955589

[bib61] Szklarczyk D, Kirsch R, Koutrouli M, Nastou K, Mehryary F, Hachilif R, Gable AL, Fang T, Doncheva NT, Pyysalo S, (2023) The STRING database in 2023: Protein-protein association networks and functional enrichment analyses for any sequenced genome of interest. Nucleic Acids Res 51: D638–D646. 10.1093/nar/gkac1000 36370105 PMC9825434

[bib62] Uhlen M, Oksvold P, Fagerberg L, Lundberg E, Jonasson K, Forsberg M, Zwahlen M, Kampf C, Wester K, Hober S, (2010) Towards a knowledge-based human protein atlas. Nat Biotechnol 28: 1248–1250. 10.1038/nbt1210-1248 21139605

[bib63] Wanders RJA, Waterham HR, Ferdinandusse S (2016) Metabolic interplay between peroxisomes and other Subcellular organelles including mitochondria and the endoplasmic reticulum. Front Cell Dev Biol 3: 83. 10.3389/fcell.2015.00083 26858947 PMC4729952

[bib64] Wanders RJA, Baes M, Ribeiro D, Ferdinandusse S, Waterham HR (2023) The physiological functions of human peroxisomes. Physiol Rev 103: 957–1024. 10.1152/physrev.00051.2021 35951481

[bib65] Waterham HR, Ferdinandusse S, Wanders RJA (2015) Human disorders of peroxisome metabolism and biogenesis. Biochim Biophys Acta Mol Cell Res 1863: 922–933. 10.1016/j.bbamcr.2015.11.015 26611709

[bib66] Woidy M, Muntau AC, Gersting SW (2018) Inborn errors of metabolism and the human interactome : A systems medicine approach. J Inherit Metab Dis 41: 285–296. 10.1007/s10545-018-0140-0 29404805 PMC5959957

[bib67] Xu W, Yan J, Shao A, Lenahan C, Gao L, Wu H, Zheng J, Zhang J, Zhang JH (2024) Peroxisome and pexophagy in neurological diseases. Fundam Res 4: 1389–1397. 10.1016/j.fmre.2023.04.016 39734532 PMC11670711

[bib68] Yang X, Coulombe-Huntington J, Kang S, Sheynkman GM, Hao T, Richardson A, Sun S, Yang F, Shen YA, Murray RR, (2016) Widespread expansion of protein interaction Capabilities by Alternative Splicing. Cell 164: 805–817. 10.1016/j.cell.2016.01.029 26871637 PMC4882190

[bib69] Yifrach E, Fischer S, Oeljeklaus S, Schuldiner M, Zalckvar E, Warscheid B (2018) Defining the mammalian peroxisomal proteome. Subcell Biochem 89: 47–66. 10.1007/978-981-13-2233-4_2 30378018

[bib70] Zalckvar E, Schuldiner M (2022) Beyond rare disorders: A new era for peroxisomal pathophysiology. Mol Cell 82: 2228–2235. 10.1016/j.molcel.2022.05.028 35714584

[bib71] Zheng C, Xia W, Zhang J (2025) Rock inhibitors in Alzheimer’s disease. Front Aging 6: 1547883. 10.3389/fragi.2025.1547883 40182055 PMC11965611

