# Supplementary material for: Peroxisomal interactome mapping enables network-based modelling of function and disease
Source: Life Sci Alliance. 2026 Jul 1;9(9):e202503562. doi: 10.26508/lsa.202503562 (PMC13324248; doi:10.26508/lsa.202503562)
Supplement: Reviewer comments [file LSA-2025-03562_review_history.pdf]

Peroxisomal interactome mapping enables network-based modelling of function and disease.

Søren Gersting, Julia Cramer, Philipp Guder, Amelie Lotz-Havla, Barbara Wolf, Heidi Noll-Puchta, Ralf Erdmann, Ania Muntau, and Mathias Woidy
DOI: https://doi.org/10.26508/lsa.202503562

Corresponding author(s): Mathias Woidy, University Medical Center Hamburg-Eppendorf

Review Timeline:

Submission Date:	2025-11-06
Editorial Decision:	2025-12-23
Revision Received:	2026-04-24
Editorial Decision:	2026-05-26
Revision Received:	2026-06-09
Accepted:	2026-06-10

Scientific Editor: Sarita Hebbar

Transaction Report:

(Note: With the exception of the correction of typographical or spelling errors that could be a source of ambiguity, letters and reports are not edited. The original formatting of letters and referee reports may not be reflected in this compilation.)

December 23, 2025

Re: Life Science Alliance manuscript #LSA-2025-03562-T

Dr Mathias Woidy
University Medical Center Hamburg-Eppendorf
University Children's Research, UCR@Kinder-UKE
Martinistr. 52
Hamburg, Hamburg 20246
Germany

Dear Dr. Woidy,

Thank you for submitting your manuscript entitled "Peroxisomal interactome mapping enables network-based modelling of function and disease." to Life Science Alliance. We apologise for the delay in communicating our decision due to editor availability issues.

The manuscript was assessed by three expert reviewers, whose comments are appended to this letter. The reviewers commented positively on the use of iBRET in living cells for mapping the peroxisomal interactions in this study. That said, they have raised several concerns that preclude publication at this point.

Reviewer 1 and 3 raised a significant point on the design of the screen. We agree that these points must be addressed in the context of discussing related findings in the manuscript.

We encourage you to provide validation of your findings, or you must tone down your conclusions on ciliary body, and retinal specific interactions as suggested by Reviewer 1.

We agree that you must address the points raised by Reviewer 1 on statistical testing. Further we concur with both Reviewers 1 and 2 that all methodological details (wet lab, iBRET screen, and computational approaches) need to be fully described to strengthen the manuscript.

Finally, in view of LSA's policy, we concur with Reviewer 2 that the source databases must be referenced and the statement on data availability must accurate and complete.

In line with their overall assessment, we invite you to submit a revised manuscript addressing the reviewers' comments. When submitting the revision, please include a letter addressing the reviewers' comments point by point. While a rebuttal must respond to all points in some form, additional experiments to resolve these points, other than indicated above, will not be required.

I would be happy to discuss the revision in more detail via email or phone/videoconferencing. Please let me know which option you prefer, if any.

To upload the revised version of your manuscript, please log in to your account: https://lsa.msubmit.net/cgi-bin/main.plex

You will be guided to complete the submission of your revised manuscript and to fill in all necessary information. Please get in touch in case you do not know or remember your login name.

The typical timeframe for revisions is three months. Please note that papers are generally considered through only one revision cycle, so strong support from the referees on the revised version is needed for acceptance.

While you are revising your manuscript, please also attend to the below editorial points to help expedite the publication of your manuscript. Please direct any editorial questions to the journal office.

Thank you for this interesting contribution to Life Science Alliance. We hope that the comments below will prove constructive as your work progresses, and we are looking forward to receiving your revised manuscript.

Sincerely,

Sarita Hebbar, PhD
Scientific Editor
Life Science Alliance
http://www.lsajournal.org

A. THESE ITEMS ARE REQUIRED FOR REVISIONS

-- A letter addressing the reviewers' comments point by point.

-- An editable version of the final text (.DOC or .DOCX) is needed for copyediting (no PDFs).

-- High-resolution figure, supplementary figure and video files uploaded as individual files: See our detailed guidelines for preparing your production-ready images, https://www.life-science-alliance.org/authors

-- Summary blurb (enter in submission system): A short text summarizing in a single sentence the study (max. 200 characters including spaces). This text is used in conjunction with the titles of papers, hence should be informative and complementary to the title and running title. It should describe the context and significance of the findings for a general readership; it should be written in the present tense and refer to the work in the third person. Author names should not be mentioned.

-- By submitting a revision, you attest that you are aware of our payment policies found here: https://www.life-science-alliance.org/copyright-license-fee

B. MANUSCRIPT ORGANIZATION AND FORMATTING:

Full guidelines are available on our Instructions for Authors page, https://www.life-science-alliance.org/authors

We encourage our authors to provide original source data, particularly uncropped/-processed electrophoretic blots and spreadsheets for the main figures of the manuscript. If you would like to add source data, we would welcome one PDF/Excel-file per figure for this information. These files will be linked as supplementary "Source Data" files.

IMPORTANT: It is Life Science Alliance policy that if requested, original data images must be made available. Failure to provide original images upon request will result in unavoidable delays in publication. Please ensure that you have access to all original microscopy and blot data images before submitting your revision.

Reviewer #1 (Comments to the Authors (Required)):

Peroxisomal interactome mapping enables network-based modelling of function and disease. Gersting et al.

The authors present a study for mapping the human peroxisomal interactome (PI) using an automated bioluminescence resonance energy transfer (iBRET) platform. The study moves beyond static lists of proteins to dynamic interaction networks, screening 92 peroxisomal proteins from the established peroxisomal proteome list (Yifrach et al., 2018) and identifying 403 binary interactions, of which 333 have so far not been reported but likely include false-positive hits. The use of iBRET in living cells is a major strength, as it accounts for the physiological environment typically missing in yeast two-hybrid (Y2H) screens. The resulting network follows a distribution, which is biologically consistent for metabolic organelles. However, there are several concerns which should be addressed for better clarity and to improve the quality of the study so that it qualifies to serve as resource for the peroxisome community and beyond.

Major concerns:
• While the BRET cutoff methodology is described, the high number of reported interactions (333 new vs. 44 confirmed) warrants caution. The authors should discuss false-positive rates specific to membrane-bound proteins in BRET assays, as overexpression can force non-physiological proximity in the crowded peroxisomal membrane.
• The authors present a compelling contradiction to established literature. Previous pan-interactome studies (e.g., Yang et al., 2016) suggested isoforms act as distinct proteins. However, this study finds peroxisomal isoforms share significantly more partners (Jaccard Index 0.18 vs 0.08). Many peroxisomal isoforms differ in internal sequences but retain the critical C-terminal PTS1 or N-terminal PTS2 targeting signals. Therefore, their interaction with the import machinery (PEX5/PEX7) would remain conserved, driving up the Jaccard Index. The authors should explicitly analyze if the shared interactions are predominantly with import receptors (PEX proteins). If the shared partners are mostly PEX5/19, the finding is an artifact of the import mechanism rather than a functional redundancy in metabolic activity.
• The authors merged their unbiased BRET data with the "HuInteractome" (literature data) to create the ePI. They rightly note that literature data suffers from study bias - disease proteins are studied more and thus appear to have higher degrees. The authors claim their BRET screen is unbiased. However, the initial selection of 92 proteins was based on the Yifrach et al. dataset, which itself has detection biases. The experimental PI is sparse (density 0.09) compared to the literature data. When analyzing the "disease module", the authors must ensure that the module's connectivity is not driven solely by the literature-derived edges, which would circularize the argument (i.e., known disease proteins interact because they were studied together

in the literature).
• The tissue specificity data is also interesting. The authors identified that while peroxins are ubiquitously expressed, the "Tissue-preferentially expressed Proteins" (TiPs) are mostly enzymes. However, since this is a resource paper, the authors should also compare this with previous large-scale tissue proteomics studies and provide the visualizations.
• The discovery that retinal TiPs interact with the "ciliary basal body" and "centrosome" components may provide a structural explanation for peroxisomal retinopathy. The interaction between the peroxisomal interactome and the ciliary basal body is a major claim. Additional evidence should be provided.
• To elevate this paper from "computational prediction" to "biological insights" the authors should validate retina-specific interactions (e.g., between a PEX protein and a ciliary protein) using co-IP or colocalization microscopy in retinal cells.
• Key wet-lab details for iBRET (cell system, constructs, instrumentation, replicates, randomization) are missing, limiting reproducibility and raising batch/confounding risks. Several computational analyses lack multiple-testing correction and precise methodological definitions (drug proximity, GO background, null models). Data/code availability is insufficient for full replication. The author must address these major concerns improve rigor and transparency.
• Multiple testing has not been controlled across key analyses (drug proximity across thousands of drugs; tissue TiP modules across many tissues; per-tissue enrichments and centrality comparisons), inflating significance. Statistical tests often assume independence despite network dependencies (shared nodes/edges), affecting degree/centrality, Jaccard, and module-size comparisons. Power-law claims for degree distributions rely on visual fits without rigorous goodness-of-fit or model comparison versus alternatives. The authors must address this and correct for this.
• In addition, improvements throughout the manuscript in terms of figures must be made, in particular the following (but not restricted to these):

Figure 2B is too large for readers to point and compare the interactions in the heatmap. The authors should divide this figure in important clusters based on peroxisomal function.
Figure misalignment and text-table inconsistencies hinder clarity and confidence in results. Authors need to review and correct discrepancies between numbers in text and table/figures.

Reviewer #2 (Comments to the Authors (Required)):

The manuscript by Gersting et al presents a comprehensive interactome study, based on the previously published iBRET method and a subset of previously published peroxisome components.

The manuscript provides valuable new iBRET data. No confirmatory analysis with orthogonal experimental approaches was done. The experimental data is contextualised with a detailed bioinformatics analysis. While comprehensive, this analysis has some room for improvement:

IntAct and BioGrid each currently have more than 1M human interactions, not even considering the other resources. I understand the proposed reference interactome undergoes multiple filtering steps (beyond the obvious merging between sources), in particular the filtering for "true binary" interactions based on [Alonso-Lopez et al. 2019]. However, the resulting number of 142,558 reference interactions still appears low. It would be useful to have more details on this process (as supplementary material), perhaps with a workflow diagram describing the reduction of numbers in each filtering step.

The major outcome of this study is the new data, and the study builds on public data. The experimental data should be submitted to a public repository like IntAct or BioGrid, and the database and accession number referenced.

All source databases should be referenced.

Please carefully review the "Data availability" section to ensure that the detail provided allows the reader to retrieve the data used in the study, or at least understand exactly which data was used and how it was retrieved. As an example, the BioGrid link is a link to a precise file, but contains the entire BioGrid release. From the context it is likely that only the "Homo_Sapiens" file was used for the analysis, but this is not explicit. For IntAct and Mint, there is just a search link without parameters.

I have not been able to find (HuInteractome, Supplementary File 3), though I went through the provided supporting material.

Reviewer #3 (Comments to the Authors (Required)):

This is a very well-done study that provides useful information and will contribute to the field of peroxisomal biology. The data is

reported transparently and available in supplemental files.

I ask authors to provide a detailed description of how the iBRET screen was performed instead of referring to the previous non-open-access article.

The list of peroxisomal proteins by Yifrach et al, which was used by the authors, is somewhat outdated. For instance, ABCD4 is clearly not a peroxisomal protein (doi: 10.1038/srep30183). In this regard, it is surprising that ABCD4 showed interactions with intraperoxisomal proteins such as ACOX2, PRDX1, PXT1 and SOD1.

Response to reviewers

The authors wish to thank the reviewers for their comments and suggestions on our manuscript that is entitled ‚Peroxisomal interactome mapping enables network-based modelling of function and disease.‘. This response letter addresses all points raised by the reviewers including how and where the text was modified. We also uploaded the manuscript marked with ‚track changes‘.

Reviewer #1 (Comments to the Authors (Required)):

Peroxisomal interactome mapping enables network-based modelling of function and disease. Gersting et al.

The authors present a study for mapping the human peroxisomal interactome (PI) using an automated bioluminescence resonance energy transfer (iBRET) platform. The study moves beyond static lists of proteins to dynamic interaction networks, screening 92 peroxisomal proteins from the established peroxisomal proteome list (Yifrach et al., 2018) and identifying 403 binary interactions, of which 333 have so far not been reported but likely include false-positive hits. The use of iBRET in living cells is a major strength, as it accounts for the physiological environment typically missing in yeast two-hybrid (Y2H) screens. The resulting network follows a distribution, which is biologically consistent for metabolic organelles. However, there are several concerns which should be addressed for better clarity and to improve the quality of the study so that it qualifies to serve as resource for the peroxisome community and beyond.

Major concerns:

• While the BRET cutoff methodology is described, the high number of reported interactions (333 new vs. 44 confirmed) warrants caution. The authors should discuss false-positive rates

specific to membrane-bound proteins in BRET assays, as overexpression can force non-physiological proximity in the crowded peroxisomal membrane.

A: We thank the reviewer for this important and well-taken comment. We are aware of the potential for false-positive BRET signals arising from non-physiological proximity of overexpressed membrane-bound proteins, particularly in a crowded membrane environment such as the peroxisomal membrane. This issue was explicitly considered during the establishment and validation of our BRET assay.

The BRET cutoff strategy applied in this study is based on previous extensive methodological validation. Using a reference data set of interacting protein pairs and non-interacting proteins, we determined a baseline false-positive rate (FPR) of approximately 4% and a true-positive rate (TPR) of 58% (Lotz-Havla, Woidy, Guder et al 2021).

In our previously published methods paper, we further investigated a subset of interaction pairs that produced mildly elevated BRET signals close to the cutoff range (gray area). These candidates were subjected to BRET saturation curve analysis, which allowed us to discriminate specific from non-specific proximity signals. Using this approach, the TPR increased to approximately 72%; however, this increase in sensitivity was accompanied by a higher FPR of approximately 12% (Lotz-Havla, Woidy, Guder et al 2021).

In the present study, we identified 333 previously unreported interactions. Since this study was intended to be a screening study, we did not retest protein pairs with only mildly elevated BRET signals. Based on the above mentioned empirically determined FPR of approximately 12%, we estimate that on the order of ~40 of these interactions may represent false positives. We have now explicitly discussed this limitation in the Discussion section and emphasize that further validation of selected candidates using orthogonal methods will be required (page 14 line 11 – line 22).

• The authors present a compelling contradiction to established literature. Previous pan-interactome studies (e.g., Yang et al., 2016) suggested isoforms act as distinct proteins. However, this study finds peroxisomal isoforms share significantly more partners (Jaccard Index 0.18 vs 0.08). Many peroxisomal isoforms differ in internal sequences but retain the critical C-terminal PTS1 or N-terminal PTS2 targeting signals. Therefore, their interaction with the import machinery (PEX5/PEX7) would remain conserved, driving up the Jaccard Index. The authors should explicitly analyze if the shared interactions are predominantly with import receptors (PEX proteins). If the shared partners are mostly PEX5/19, the finding is an artifact of the import mechanism rather than a functional redundancy in metabolic activity.

A: The reviewer is right. Our result could be an artefact of the import machinery (PEX5, PEX7 and PEX19). That said, after removing PPIs with PEX19, PEX5 and PEX7, the JI is 0.10 for isoform pairs and 0.04 for all other non-isoform pairs. The JI still differs significantly (P-value = 0.014, Wilcoxon-rank test). This indicates that our finding could be more than just an artifact of the import mechanism. We have added this to the Results (page 6 line 25 – page 7 line 3).

• The authors merged their unbiased BRET data with the "HuInteractome" (literature data) to create the ePI. They rightly note that literature data suffers from study bias - disease proteins are studied more and thus appear to have higher degrees. The authors claim their BRET screen is unbiased. However, the initial selection of 92 proteins was based on the Yifrach et al. dataset, which itself has detection biases. The experimental PI is sparse (density 0.09) compared to the literature data. When analyzing the "disease module", the authors must ensure that the module's connectivity is not driven solely by the literature-derived edges, which would circularize the argument (i.e., known disease proteins interact because they were studied together in the literature).

A: Indeed, the selection of the proteins for our BRET screen was based on the Yifrach et al. dataset. In that way, the selection is biased to include proteins that are associated with peroxisomes, which Yifrach et al indicated via their localization score. However, that was the purpose of the study, and we indicate the number of proteins with low or high detection frequency in Fig1A.

We would like to clarify that our Literature Interactome (HuInteractome) is actually very sparse: considering only the previously known peroxisomal interactions, the network density is 0.02, and when considering the complete HuInteractome, the density drops to 0.001 (142558/124685737 = 0.001). This is substantially lower than the density of our experimental BRET-derived PI (0.09), strongly reducing the likelihood that the observed disease module connectivity is driven solely by literature edges.

To further address the reviewer's concern, we analyzed the disease module using only BRET-derived edges. In this network, 43 of the 48 disease-associated proteins remain connected through 143 BRET-derived interactions. In contrast, a disease module constructed solely from literature-derived PPIs connects only 33 of the 88 disease-associated proteins with 60 edges. These results indicate that the connectivity of the disease module is largely supported by the experimentally derived BRET interactions rather than being driven by literature-derived edges. We have added this to the Results (page 9 lines 1 – 6).

• The tissue specificity data is also interesting. The authors identified that while peroxins are ubiquitously expressed, the "Tissue-preferentially expressed Proteins" (TiPs) are mostly enzymes. However, since this is a resource paper, the authors should also compare this with previous large-scale tissue proteomics studies and provide the visualizations.

A: The reviewer raises a valid point: it would indeed be interesting to assess whether our findings are specific to peroxisomes or extend to other organelles. To address this, we performed an additional analysis using lysosomes as a representative example. We curated a list of lysosomal enzymes and proteins involved in lysosomal biogenesis (n_biogenesis = 174; n_enzymes = 73).

We then compared the frequency of tissue-preferential expression (TiP) between these two groups. Lysosomal proteins involved in biogenesis did not differ significantly from lysosomal enzymes in their tissue specificity (mean_biogenesis = 2.97 vs. mean_enzymes = 3.05; Wilcoxon test, $p = 0.55$).

• The discovery that retinal TiPs interact with the "ciliary basal body" and "centrosome" components may provide a structural explanation for peroxisomal retinopathy. The interaction between the peroxisomal interactome and the ciliary basal body is a major claim. Additional evidence should be provided.

A: We thank the reviewer for highlighting this point. We agree that establishing a direct mechanistic link between the peroxisomal interactome and components of the ciliary basal body or centrosome would require additional experimental evidence. Our intention was not to present this observation as a definitive mechanistic conclusion, but rather to illustrate how the interaction dataset generated in this study can be explored to identify potentially relevant connections between peroxisomal proteins and tissue-specific pathways.

The example of retinal tissue-preferential proteins (TiPs) interacting with proteins associated with the ciliary basal body or centrosome was therefore included primarily to demonstrate how the dataset may be used to generate biologically meaningful hypotheses that could guide future studies. We agree that these observations should be interpreted with appropriate caution and

do not by themselves establish a causal relationship between peroxisomal protein interactions and retinal pathology.
To clarify this point, we have added a statement in the Discussion (see also next point).

• To elevate this paper from "computational prediction" to "biological insights" the authors should validate retina-specific interactions (e.g., between a PEX protein and a ciliary protein) using co-IP or colocalization microscopy in retinal cells.

A: The reviewer raises an important point. The primary aim of the present study is to expand the peroxisomal interactome and to demonstrate how these newly generated interaction data can be integrated with computational analyses to identify potential functional links and generate testable hypotheses. While the retina-associated interactions highlighted here are intriguing, the focus of this work is on systematic network expansion and resource generation rather than on the experimental validation of individual interaction pairs in specific cellular contexts.
We agree that validation of selected interactions, for example by co-immunoprecipitation or colocalization analyses in retinal cells, would provide valuable biological insight. However, performing such targeted experiments would require dedicated model systems and additional experimental frameworks that go beyond the scope of the present study. We therefore consider the interactions identified here as hypotheses that can guide future experimental investigations.
To clarify this point, we have added a statement in the Discussion emphasizing that the predicted retina-associated interactions represent candidates for future functional validation (page 17 lines 8 – 22).

• Key wet-lab details for iBRET (cell system, constructs, instrumentation, replicates, randomization) are missing, limiting reproducibility and raising batch/confounding risks.

A: The reviewer is right and we have added a detailed description of our iBRET procedure in the Methods section (page 19 line 3 – page 21 line 19).

Several computational analyses lack multiple-testing correction and precise methodological definitions (drug proximity, GO background, null models). Data/code availability is insufficient for full replication. The author must address these major concerns improve rigor and transparency.

A: We thank the reviewer for this important comment. We added information about the background used for GO, drugtarget and disease gene enrichment analysis in the respective method section. Furthermore, we adapted our null model to degree preserved random drawing throughout the paper. This changed the z-scores slightly however no main result was altered. To better assess significance, we calculated an empirical p value for LCC comparisons and corrected it with Bonferroni correction where applicable. In addition, we now also provide the links to the GitHub repositories for the Python and R code.
For further details on adaptions of the analyses, please see also our answer to the next question.

• Multiple testing has not been controlled across key analyses (drug proximity across thousands of drugs; tissue TiP modules across many tissues; per-tissue enrichments and centrality comparisons), inflating significance. Statistical tests often assume independence despite network dependencies (shared nodes/edges), affecting degree/centrality, Jaccard, and module-size comparisons. Power-law claims for degree distributions rely on visual fits without rigorous goodness-of-fit or model comparison versus alternatives. The authors must address this and correct for this.

A: We agree that multiple testing and network dependencies should be carefully considered when interpreting results derived from large interaction networks. For the drug proximity analysis, we followed the commonly used network-based proximity framework in which the proximity between drug targets and a disease module is quantified and normalized using z-scores derived from degree-preserving randomizations of the network (Guney et al., 2016). In this approach, the z-score serves as a relative measure of network proximity that allows drugs to be ranked according to their distance to the disease module, rather than as a classical hypothesis-testing framework across independent statistical tests.

Consistent with previous studies using this methodology, the drug proximity results were therefore primarily interpreted as a ranking of candidate compounds based on their relative network distance. To avoid overinterpretation, we have removed the term "significant" from the Results section and now present the drug proximity results as relative rankings based on their z-scores (page 9 line 16 - 17).

Similarly, the analyses of tissue-specific TiP modules and tissue-level enrichments were intended as exploratory network analyses aimed at identifying patterns rather than performing formal hypothesis testing across independent samples. We acknowledge that network-based analyses inherently involve dependencies between nodes and edges, which can affect classical statistical assumptions. To mitigate such biases, several of the analyses rely on comparisons to randomized networks. We adapted these so all analyses involving random comparisons to preserve now a key network property "node degree", thereby providing an appropriate reference framework for evaluating the observed patterns. Despite only providing the z-scores we have now also calculated an empirical P-value for lcc comparisons and corrected it with Bonferroni correction. Following this, we now only show 9 significant (corrected $p < 0.05$) TiP modules and adopted this in the Results section and Figure 3 accordingly.

Regarding the degree distribution, we agree that rigorous statistical comparison of alternative models would be required to formally demonstrate a power-law distribution. Therefore, we have

extended the analysis to include a formal goodness-of-fit test and explicit model comparison. A discrete power-law model was fitted using the poweRlaw package (Version 1.0.0) for R and evaluated by a bootstrap-based Kolmogorov–Smirnov test (function bootstrap_p, 1000 sims), which did not reject the model (PI: P-value = 0.112; ePI P-value = 0.325). A likelihood-ratio test (function compare_distributions) comparing the power-law and log-normal models yielded a negative but non-significant result (PI: R = −0.680, P-value = 0.496; ePI R = -0.250, P-value = 0.802), indicating no clear preference for either distribution. The manuscript has been revised accordingly to avoid strong claims of scale-free behavior (page 5 line 23 – page 6 line 2 & page 7 lines 15 - 16). We have adopted Methods accordingly (page 22 line 3 – line 9).

• In addition, improvements throughout the manuscript in terms of figures must be made, in particular the following (but not restricted to these): Figure 2B is too large for readers to point and compare the interactions in the heatmap. The authors should divide this figure in important clusters based on peroxisomal function. Figure misalignment and text-table inconsistencies hinder clarity and confidence in results. Authors need to review and correct discrepancies between numbers in text and table/figures.

A: We thank the reviewer for these helpful suggestions regarding figure clarity and consistency. To address the concern about Figure 2B being too large and difficult to interpret, we have removed this panel from the main figure and now provide it as Supplementary Figure S3. This allows us to improve the readability and overall layout of Figure 2, while still making the full interaction heatmap available for interested readers in the supplementary material.

In addition, we have carefully reviewed all figures, tables, and corresponding descriptions in the manuscript to correct misalignments and ensure consistency between reported values in the text and those shown in figures and tables. These revisions improve the clarity and reliability of the presented results.

Reviewer #2 (Comments to the Authors (Required)):

The manuscript by Gersting et al presents a comprehensive interactome study, based on the previously published iBRET method and a subset of previously published peroxisome components.

The manuscript provides valuable new iBRET data. No confirmatory analysis with orthogonal experimental approaches was done. The experimental data is contextualised with a detailed bioinformatics analysis. While comprehensive, this analysis has some room for improvement:

IntAct and BioGrid each currently have more than 1M human interactions, not even considering the other resources. I understand the proposed reference interactome undergoes multiple filtering steps (beyond the obvious merging between sources), in particular the filtering for "true binary" interactions based on [Alonso-Lopez et al. 2019]. However, the resulting number of 142,558 reference interactions still appears low. It would be useful to have more details on this process (as supplementary material), perhaps with a workflow diagram describing the reduction of numbers in each filtering step.

A: *We generated a flowchart diagram summarizing all processing steps and the corresponding number of interactions retained at each stage. This diagram has been added to the supplementary material (Supplemental Figure S7). It is correct that, already in 2023 when the data were retrieved, both IntAct and BioGRID contained more than 800,000 interactions. Restricting the datasets to human interactions substantially reduced the number of interactions, particularly for IntAct, whereas the numbers in BioGRID and HuRI were only marginally affected. Identifier translation further reduced the number of interactions across all databases except BioGRID, which already provides interactions in GeneID format; however, this step had only a minor overall impact. The largest reduction in interaction counts occurred during the filtering step restricting the dataset to binary interactions.*

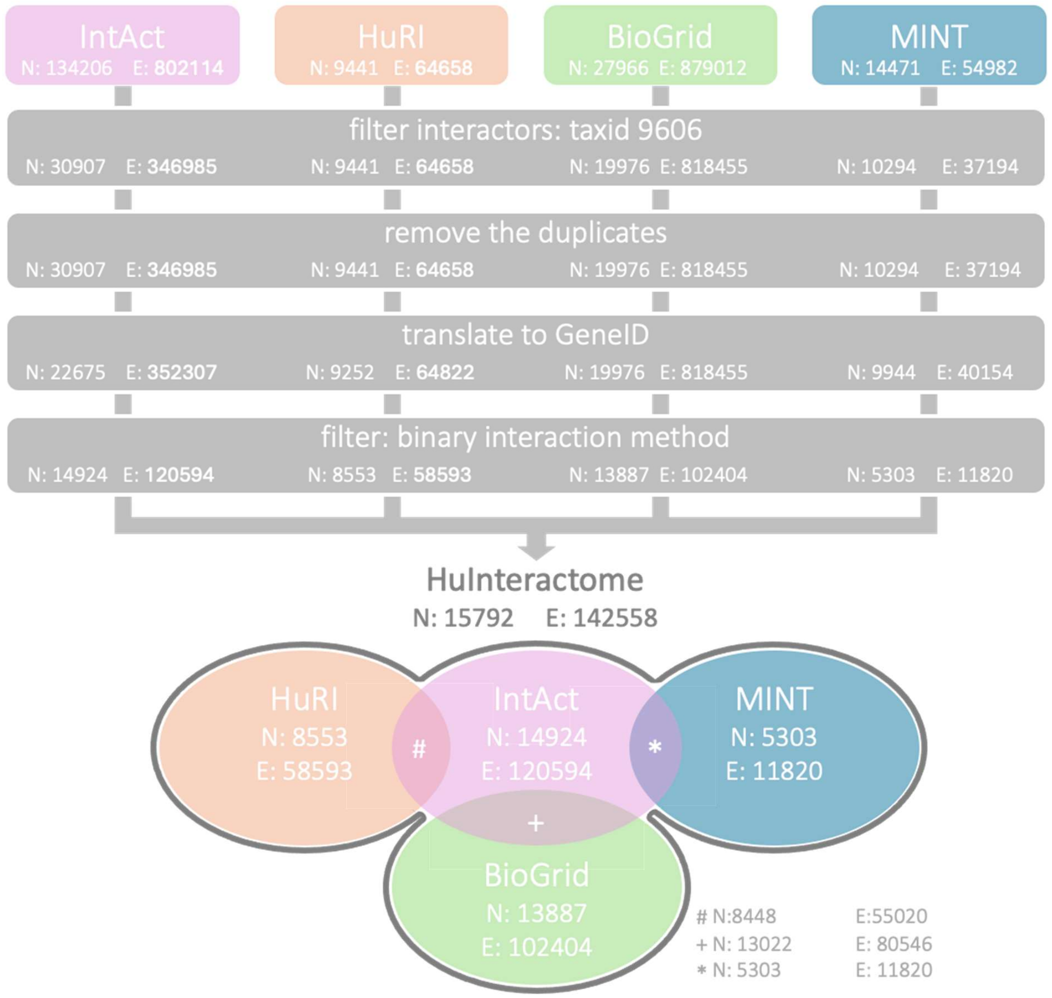

Supplemental Figure S7. Flowchart presenting the data processing steps applied to generate the HuInteractome. *Flowchart presenting the data processing steps applied to generate the HuInteractome. The databases IntAct, HuRI, BioGrid and MINT were separately filtered to contain only interactions where both interactors are tagged with taxid 9606, followed by removing duplicated entries. Then, the protein names were translated to GeneID: if one protein name mapped to several GeneIDs, all GeneIDs were added; if no translation was found, the entry was deleted. In the last step, we filtered the resulting interactions for binary interaction methods. N= nodes and E = edges of a graph containing all entries of the current processing step.*

The major outcome of this study is the new data, and the study builds on public data. The experimental data should be submitted to a public repository like IntAct or BioGrid, and the database and accession number referenced.

A: Thank you for this helpful suggestion. We agree that making the data publicly accessible is important. The experimental data generated in this study have now been submitted to BioGRID (https://wiki.thebiogrid.org/doku.php/gersting2026), and we will include the corresponding link in the revised manuscript. As noted, our study also builds on previously published public datasets, which are appropriately referenced.

All source databases should be referenced. Please carefully review the "Data availability" section to ensure that the detail provided allows the reader to retrieve the data used in the study, or at least understand exactly which data was used and how it was retrieved. As an example, the BioGrid link is a link to a precise file, but contains the entire BioGrid release. From the context it is likely that only the "Homo_Sapiens" file was used for the analysis, but this is not explicit. For IntAct and Mint, there is just a search link without parameters.

A: Thank you very much for this comment. We carefully went through all links provided, changed them to directly link to the version used or added version details where applicable. Additionally, we added the query for the PSICQUIC which pointed to the database release 2023-06-02 at 26th of August 2023.

I have not been able to find (HuInteractome, Supplementary File 3), though I went through the provided supporting material.

A: We are sorry for this inconvenience. This has been fixed.

Reviewer #3 (Comments to the Authors (Required)):

This is a very well-done study that provides useful information and will contribute to the field of peroxisomal biology. The data is reported transparently and available in supplemental files. I ask authors to provide a detailed description of how the iBRET screen was performed instead of referring to the previous non-open-access article.

A: We have added detailed description of the iBRET process in the Methods section (page 19 line 3 – page 21 line 19).

The list of peroxisomal proteins by Yifrach et al, which was used by the authors, is somewhat outdated. For instance, ABCD4 is clearly not a peroxisomal protein (doi: 10.1038/srep30183). In this regard, it is surprising that ABCD4 showed interactions with intraperoxisomal proteins such as ACOX2, PRDX1, PXT1 and SOD1.

A: We thank the reviewer for pointing this out. To define the set of peroxisomal proteins used in our analyses, we relied on the dataset reported by Yifrach et al. in order to apply an established and systematic resource and to avoid introducing subjective bias when compiling the list of peroxisomal proteins.

We agree that more recent studies indicate that ABCD4 is not localized to peroxisomes but rather to lysosomes. To assess whether the inclusion of ABCD4 influenced our results, we repeated the relevant analyses after excluding ABCD4 from the dataset. This reanalysis did not lead to any changes in the overall results or conclusions (see below).

For transparency and consistency with the original dataset used to define the peroxisomal protein set, we therefore retained ABCD4 in the manuscript while acknowledging that its peroxisomal localization is currently considered unlikely.

Reanalysis without ABCD4:

- *Number of nodes: 172; Number of edges: 439; Average degree: 5.1047*
- *ABCD4 is never a TiP in any tissue so it should not influence the TiP results.*
- *ABCD4 is no homooligomer.*
- *ABCD4 is a disease gene, LCC without ABCD4 is 55 (original 56), LCC of the disease genes is greater than average random sample. Z-score: 17.783 LCC: 55 P-value 0.0009990*

May 26, 2026

Re: Life Science Alliance manuscript #LSA-2025-03562-TR

Dr. Mathias Woidy
University Medical Center Hamburg-Eppendorf
University Children's Research, UCR@Kinder-UKE
Martinistr. 52
Hamburg, Hamburg 20246
Germany

Dear Dr. Woidy,

Thank you for submitting your manuscript entitled "Peroxisomal interactome mapping enables network-based modelling of function and disease." to Life Science Alliance.

Your revised manuscript was reviewed by all the original reviewers. The reviewers have acknowledged that your revisions have largely addressed their concerns. However Reviewer 3 has reiterated a valid point that is pending, i.e., resources must be appropriately and accurately referenced. We agree with this reviewer that you must resolve these relevant points with minor yet pertinent additions in the manuscript text.
Kindly move the relevant statements from 'Data and Code' availability to the general section on 'Data Availability' as per LSA guidelines.

Overall, we would be happy to publish your paper in Life Science Alliance pending resolution of the reviewers' requests and final changes necessary to meet our formatting guidelines.

-Please add the X and Bluesky handles of your host institute/organization, as well as your own, and/or one of the authors, in our system
-Please remove Word / Character Count from the title page
-Please rename "Competing interests" to "Conflict of Interest."
-Please use the [10 author names et al.] format in your references (i.e., limit the author names to the first 10)

I would be happy to discuss the revision in more detail via email or phone/videoconferencing. Please let me know which option you prefer, if any.

To upload the revised version of your manuscript, please log in to your account: https://lsa.msubmit.net/cgi-bin/main.plex

You will be guided to complete the submission of your revised manuscript and to fill in all necessary information. Please get in touch in case you do not know or remember your login name.

The typical timeframe for revisions is three months. Please note that papers are generally considered through only one revision cycle, so strong support from the referees on the revised version is needed for acceptance.

While you are revising your manuscript, please also attend to the below editorial points to help expedite the publication of your manuscript. Please direct any editorial questions to the journal office. When submitting the revision, please include a letter addressing the reviewers' comments point by point.

Thank you for this interesting contribution to Life Science Alliance. We hope that the comments below will prove constructive as your work progresses, and we are looking forward to receiving your revised manuscript.

Sincerely,

Sarita Hebbar, PhD
Scientific Editor
Life Science Alliance
http://www.lsajournal.org

A. THESE ITEMS ARE REQUIRED FOR REVISIONS

-- A letter addressing the reviewers' comments point by point.

-- An editable version of the final text (.DOC or .DOCX) is needed for copyediting (no PDFs).

-- High-resolution figure, supplementary figure and video files uploaded as individual files: See our detailed guidelines for preparing your production-ready images, https://www.life-science-alliance.org/authors

-- Summary blurb (enter in submission system): A short text summarizing in a single sentence the study (max. 200 characters including spaces). This text is used in conjunction with the titles of papers, hence should be informative and complementary to the title and running title. It should describe the context and significance of the findings for a general readership; it should be written in the present tense and refer to the work in the third person. Author names should not be mentioned.

-- By submitting a revision, you attest that you are aware of our payment policies found here: https://www.life-science-alliance.org/copyright-license-fee

B. MANUSCRIPT ORGANIZATION AND FORMATTING:

Full guidelines are available on our Instructions for Authors page, https://www.life-science-alliance.org/authors

We encourage our authors to provide original source data, particularly uncropped/-processed electrophoretic blots and spreadsheets for the main figures of the manuscript. If you would like to add source data, we would welcome one PDF/Excel-file per figure for this information. These files will be linked as supplementary "Source Data" files.

IMPORTANT: It is Life Science Alliance policy that if requested, original data images must be made available. Failure to provide original images upon request will result in unavoidable delays in publication. Please ensure that you have access to all original microscopy and blot data images before submitting your revision.

Reviewer #1 (Comments to the Authors (Required)):

The authors have adequately addressed all points raised. The revised version of the paper can be published in its present form.

Reviewer #2 (Comments to the Authors (Required)):

The authors have addressed some of my concerns, but the resource referencing still needs to be improved:

In addition to the (now mostly correct) data references, please cite all database resources with their appropriate literature references (typically most recent NAR DB issue) where the resource name first appears in the text. This includes GO, UniProt, IntAct, PSICQUIC, BioGrid, STRING, MINT, HGNC, NCBI (ideally to the correct actual database), Human Protein Atlas, perhaps BIND, HPRD (see comment below), I might have forgotten some.

There are two sections "Data and Code availability" and "Data availability", with different contents, which is confusing. This might be due to prescribed manuscript structure, and might be resolved by merging both, and referencing from "Data availability" back to the comprehensive "Data and Code availability" section.

Line 497 "Curation of reference data sets (PRS and RRS)":
These two sets are not referenced to supplementary material or Zenodo, and I could not find the data files. Please provide. Also, did you really extract data from BIND? This resource has not been available as a maintained resource for more than ten years. Similarly, I have not been able to access HPRD for a while?

Missing blank in line 599

Line 684 has a formatting error merging the IntAct entry to the previous item.

Reviewer #3 (Comments to the Authors (Required)):

Using BRET screen, authors map peroxisomal interactome. Authors used transcriptomic data to predict tissues specific interactomes, did GO analysis of non-peroxisomal interaction and identified potential drug targets. The experimental data is

properly integrated and interpretated with bioinformatics. In the revised version of the manuscript authors more properly describe the experimental methodology and filtering steps in the data processing. Overall, the manuscript provides excellent hypothesis-generating datasets which can help with future peroxisomal research. I don't see any other issues that would have to be adressed.

Response to reviewers

The authors wish to thank the reviewers for their comments and suggestions on our manuscript that is entitled ‚Peroxisomal interactome mapping enables network-based modelling of function and disease.'. This response letter addresses the points raised by the reviewers including how and where the text was modified.

Reviewer #1 (Comments to the Authors (Required)):

The authors have adequately addressed all points raised. The revised version of the paper can be published in its present form.

A: We thank the reviewer for the positive assessment of our revised manuscript and for the constructive feedback provided during the review process.

Reviewer #2 (Comments to the Authors (Required)):

The authors have addressed some of my concerns, but the resource referencing still needs to be improved:

In addition to the (now mostly correct) data references, please cite all database resources with their appropriate literature references (typically most recent NAR DB issue) where the resource name first appears in the text. This includes GO, UniProt, IntAct, PSICQUIC, BioGrid, STRING, MINT, HGNC, NCBI (ideally to the correct actual database), Human Protein Atlas, perhaps BIND, HPRD (see comment below), I might have forgotten some.

A: We have carefully revised the manuscript and added the appropriate literature references recommended by the respective database providers at the first occurrence of each resource in the text. This includes, among others, GO, UniProt, IntAct, PSICQUIC, BioGRID, STRING, MINT, HGNC, NCBI resources, and the Human Protein Atlas. References for BIND and HPRD

have also been added where these resources are mentioned in the context of the historical compilation of the reference dataset.

There are two sections "Data and Code availability" and "Data availability", with different contents, which is confusing. This might be due to prescribed manuscript structure, and might be resolved by merging both, and referencing from "Data availability" back to the comprehensive "Data and Code availability" section.

A: We have merged both sections into "Data and Code availability".

Line 497 "Curation of reference data sets (PRS and RRS)":
These two sets are not referenced to supplementary material or Zenodo, and I could not find the data files. Please provide. Also, did you really extract data from BIND? This resource has not been available as a maintained resource for more than ten years. Similarly, I have not been able to access HPRD for a while?

A: We thank the reviewer for this comment. Indeed, the reference datasets used for threshold definition were not previously deposited in a public repository but were described in our earlier publication (Lotz-Havla, Woidy, Guder et al., 2021). To improve transparency and reproducibility, we have now deposited the raw datasets used for threshold determination in Zenodo and added the corresponding reference in the revised manuscript (page 21 line 20).
The reviewer is correct that the BIND and HPRD databases are no longer actively maintained and are not readily accessible today. The reference dataset was originally compiled several years ago using a combination of literature curation and searches of publicly available interaction databases, including BIND and HPRD when these resources were still accessible. To clarify the evidence underlying the PPIs included in the reference dataset, we now provide

a comprehensive list of the corresponding literature sources. This evidence table has also been deposited in Zenodo.

Missing blank in line 599

A: Thank you. We found several other occasions with missing blanks and have corrected these.

Line 684 has a formatting error merging the IntAct entry to the previous item.

A: Thank you, we have fixed it.

Reviewer #3 (Comments to the Authors (Required)):

Using BRET screen, authors map peroxisomal interactome. Authors used transcriptomic data to predict tissues specific interactomes, did GO analysis of non-peroxisomal interaction and identified potential drug targets. The experimental data is properly integrated and interpretated with bioinformatics. In the revised version of the manuscript authors more properly describe the experimental methodology and filtering steps in the data processing. Overall, the manuscript provides excellent hypothesis-generating datasets which can help with future peroxisomal research. I don't see any other issues that would have to be adressed.

A: We thank the reviewer for the positive evaluation and are pleased that the revisions have satisfactorily addressed all concerns.

June 10, 2026

RE: Life Science Alliance Manuscript #LSA-2025-03562-TRR

Dr. Mathias Woidy
University Medical Center Hamburg-Eppendorf
University Children's Research, UCR@Kinder-UKE
Martinistr. 52
Hamburg, Hamburg 20246
Germany

Dear Dr. Woidy,

Thank you for submitting your Research Article entitled "Peroxisomal interactome mapping enables network-based modelling of function and disease". It is a pleasure to let you know that your manuscript is now accepted for publication in Life Science Alliance. Congratulations on this interesting work.

The final published version of your manuscript will be deposited by us to PubMed Central upon publication.

Your manuscript will now progress through copyediting and proofing. It is journal policy that authors provide original data upon request.

Reviews, decision letters, and point-by-point responses associated with peer-review at Life Science Alliance will be published, alongside the manuscript. If you do want to opt out of having the reviewer reports and your point-by-point responses displayed, please let us know immediately.

IMPORTANT: If you will be unreachable at any time, please provide us with the email address of an alternate author. Failure to respond to routine queries may lead to unavoidable delays in publication.

Scheduling details will be available from our production department. You will receive proofs shortly before the publication date. Only essential corrections can be made at the proof stage so if there are any minor final changes you wish to make to the manuscript, please let the journal office know now.

DISTRIBUTION OF MATERIALS:
Authors are required to distribute freely any materials used in experiments published in Life Science Alliance. Authors are encouraged to deposit materials used in their studies to the appropriate repositories for distribution to researchers.

You can contact the journal office with any questions, contact@life-science-alliance.org

Again, congratulations on a very nice paper. I hope you found the review process to be constructive and are pleased with how the manuscript was handled editorially. We look forward to future exciting submissions from your lab.

Sincerely,

Sarita Hebbar, PhD
Scientific Editor
Life Science Alliance
http://www.lsajournal.org
